# Multi-seasonal systematic camera-trapping reveals fluctuating densities and high turnover rates of Carpathian lynx on the western edge of its native range

**DOI:** 10.1038/s41598-021-88348-8

**Published:** 2021-04-29

**Authors:** Martin Duľa, Michal Bojda, Delphine B. H. Chabanne, Peter Drengubiak, Ľuboslav Hrdý, Jarmila Krojerová-Prokešová, Jakub Kubala, Jiří Labuda, Leona Marčáková, Teresa Oliveira, Peter Smolko, Martin Váňa, Miroslav Kutal

**Affiliations:** 1 Department of Forest Ecology, Faculty of Forestry and Wood Technology, Mendel University in Brno, Zemědělská 1, 613 00 Brno, Czech Republic; 2 Friends of the Earth Czech Republic, Olomouc Branch, Dolní náměstí 38, 779 00 Olomouc, Czech Republic; 3 Centre for Sustainable Aquatic Ecosystems, Harry Butler Institute, Murdoch University, Murdoch, WA Australia; 4 Evolutionary Genetics Group, Department of Anthropology, University of Zurich, Zurich, Switzerland; 5 Kysuce Protected Landscape Area Administration, State Nature Conservancy of the Slovak Republic, U Tomali č. 1511, 022 01 Čadca, Slovakia; 6 Institute of Vertebrate Biology of the Czech Academy of Sciences, Květná 8, 603 65 Brno, Czech Republic; 7 Department of Zoology, Fisheries, Hydrobiology and Apiculture, Faculty of AgriSciences, Mendel University in Brno, Zemědělská 1, 613 00 Brno, Czech Republic; 8 Department of Forestry and Renewable Forest Resources, Biotechnical Faculty, University of Ljubljana, Jamnikarjeva 101, 1000 Ljubljana, Slovenia; 9 Department of Applied Zoology and Wildlife Management, Faculty of Forestry, Technical University in Zvolen, T. G. Masaryka 24, 960 01 Zvolen, Slovakia; 10 DIANA – Carpathian Wildlife Research, Mládežnícka 47, 974 04 Banská Bystrica, Slovakia

**Keywords:** Biodiversity, Conservation biology, Population dynamics, Ecology, Zoology

## Abstract

Camera-trapping and capture-recapture models are the most widely used tools for estimating densities of wild felids that have unique coat patterns, such as Eurasian lynx. However, studies dealing with this species are predominantly on a short-term basis and our knowledge of temporal trends and population persistence is still scarce. By using systematic camera-trapping and spatial capture-recapture models, we estimated lynx densities and evaluated density fluctuations, apparent survival, transition rate and individual's turnover during five consecutive seasons at three different sites situated in the Czech–Slovak–Polish borderland at the periphery of the Western Carpathians. Our density estimates vary between 0.26 and 1.85 lynx/100 km^2^ suitable habitat and represent the lowest and the highest lynx densities reported from the Carpathians. We recorded 1.5–4.1-fold changes in asynchronous fluctuated densities among all study sites and seasons. Furthermore, we detected high individual’s turnover (on average 46.3 ± 8.06% in all independent lynx and 37.6 ± 4.22% in adults) as well as low persistence of adults (only 3 out of 29 individuals detected in all seasons). The overall apparent survival rate was 0.63 ± 0.055 and overall transition rate between sites was 0.03 ± 0.019. Transition rate of males was significantly higher than in females, suggesting male-biased dispersal and female philopatry. Fluctuating densities and high turnover rates, in combination with documented lynx mortality, indicate that the population in our region faces several human-induced mortalities, such as poaching or lynx-vehicle collisions. These factors might restrict population growth and limit the dispersion of lynx to other subsequent areas, thus undermining the favourable conservation status of the Carpathian population. Moreover, our study demonstrates that long-term camera-trapping surveys are needed for evaluation of population trends and for reliable estimates of demographic parameters of wild territorial felids, and can be further used for establishing successful management and conservation measures.

## Introduction

Knowledge of demographic parameters of a population is fundamental for the successful conservation and management of many species, especially endangered ones^1^. Regarding large carnivores, population size estimation represents a difficult task owing to their large home ranges, low densities and cryptic nature e.g.^2–5^. Recent development of digital camera traps has triggered research on elusive carnivores^3^ and enabled conventional and spatially explicit capture–recapture modelling methods to become common tools for estimating demographic parameters of many wild felids that have unique coat patterns^6^.

The Eurasian lynx (*Lynx lynx*), an umbrella species and the flagship of predator recovery efforts throughout Europe e.g.^7^, represents a suitable model species for camera-trapping surveys^8–11^. At present, the Eurasian lynx is a fully protected species in most European countries and its conservation is further enforced by the EU’s Wild Flora and Fauna Habitats Directive, aiming for “favourable conservation status” of the population. Despite the relatively positive status of European native populations (e.g. Carelian, Baltic or Carpathian), they are likely to be threatened to varying degrees by traffic accidents, habitat fragmentation, conflicts with hunters and, to a lesser extent, with livestock breeders. These conflicts give rise to a negative attitude towards lynx conservation and often lead to retaliation in the form of illegal acts, representing the main threats for the lynx in many areas^12^.

Successful plans for conservation and management of lynx populations across Europe should rely on robust demographic data. Although the abundance and population density of several reintroduced populations (e.g., in the Swiss Alps, French Jura and in the Bavarian Forest) have been intensively studied^8–10, 13^, the status of their source, the Carpathian population, is based mainly on rough national estimates that are challenged by a few local studies as having been overestimated^11, 14^.

For twenty years (1970s–1990s), the Carpathian population became a source for successful lynx reintroductions into several areas in central, western and southern Europe^15, 16^. In addition, more animals are currently being captured in the Carpathians and translocated within the reinforcement and reintroduction lynx projects in Dinaric Mts, Slovenia and Palatine Forest, Germany (https://www.lifelynx.eu/ and https://snu.rlp.de/de/projekte/luchs/, respectively). This highlights the necessity to obtain robust demographic data about this native population.

Even though a noticeable lack of scientific involvement was considered the main constraint for lynx management in the Carpathians 17 years ago^17^, only a few studies based on short-term camera-trapping have been conducted since that time^11, 18–20^. Likewise, at the pan-European scale, most of the published density estimates are based only on short-term camera-trapping surveys conducted within one or two seasons e.g.^9, 13^. However, long-term studies conducted on other felids, e.g. tigers, revealed significant annual fluctuations in densities^21, 22^ or in the turnover rate^23^. Indeed, previous research of the Alpine population also suggested that lynx density can fluctuate between years^24^.

Species abundance can also vary in space depending on several environmental variables and also the geographical position in species distribution or historical range^25^. Core areas should have higher density and lower turnover compared to the edges, according to the centre distribution hypothesis^26^ and the centre-periphery hypothesis^27^. Although demographic parameters of populations often do not follow these expectations^25^, no study thus far has investigated demographic patterns in the continuous part of Eurasian lynx distribution range, although, e.g., lynx census in Sweden and Norway revealed a substantial variation of family group densities in Scandinavian population^28^.

The aim of this study was to evaluate fluctuations in the density of the Eurasian lynx at the core–edge gradient of its distribution range in the Western Carpathians, and to assess other demographic parameters—apparent survival, transition probability and the turnover of individual lynx within the studied local populations. We expected higher population densities and higher apparent survival within the core compared to the edge. However, we hypothesized that the apparent survival would be higher and the turnover and transition rate would be lower in females (due to male-biased dispersal^29^). This study helps to fill the gap in the knowledge of the native Carpathian lynx population and provides the first multi-seasonal population dynamics data about this elusive carnivore.

## Methods

### Study area

**Figure 1:**
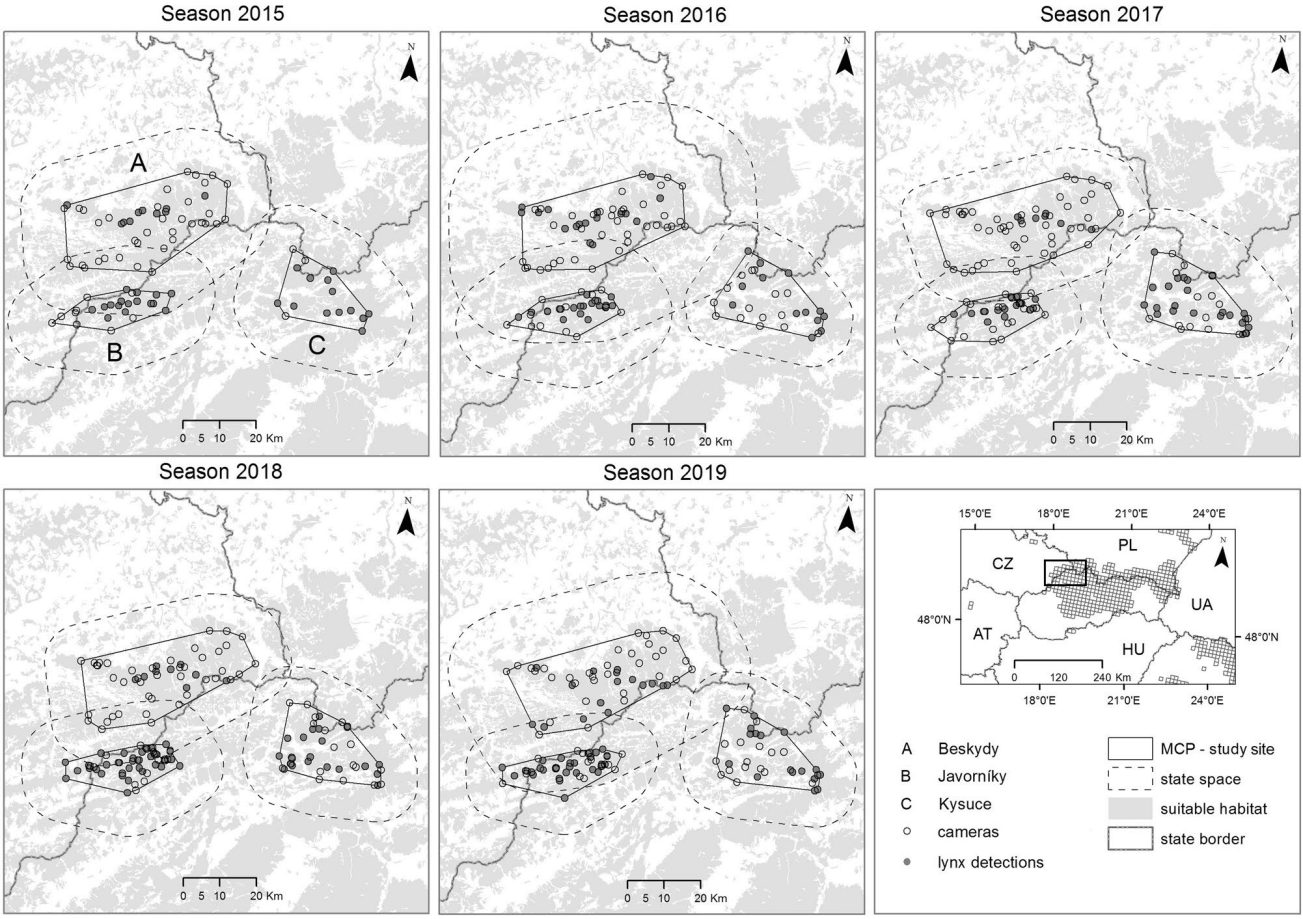
Map of the study area, particular study sites and location of cameras with lynx detections in five seasons of systematic camera-trapping in the Czech-Slovak-Polish borderland situated at the western edge of the Carpathian Mountains. Minimum convex polygons (MCP) were enlarged by buffers resulting in a state-space in which the density of lynx was estimated. The EEA squares (10 × 10 km) in the inset show the permanent lynx distribution according to Chapron et al.^62^. The figure was created in ArcMap 10.7.1 (https://desktop.arcgis.com/en/arcmap/)^43^.

The study was conducted in the Czech-Slovak-Polish borderland at the periphery of the Western Carpathians. We chose three model study sites: Beskydy, Javorníky and Kysuce (Fig. 1). The „Beskydy” site is situated at the edge of the most western range, the site „Kysuce'' is near the core of West-Carpathian lynx distribution and breeding stronghold in Slovakia^30–32^) and the site “Javorníky” is situated in the middle of this edge-core gradient (Fig. 1). Among all study sites, altitude ranges from 350 to 1 324 m a.s.l., which causes a cold mountain climate with average year temperatures from 2 to 7 degrees. Yearly mean precipitation is 800–1 400 mm, and the ground is usually covered with snow from mid-November to late March or April^33–35^. Forests cover 70% of the whole study area (1 609 km^2^) and are dominated by Norway spruce (*Picea abies*), mainly in the form of plantations, and by beech (*Fagus sylvatica*). Only small areas of natural forests are present, situated primarily in protected natural reserves. The landscape in all sites is intensively used for diverse human activities. Besides forestry and hunting practices, there are also high levels of tourism and grazing activities. Human density ranges from 80 to 192 inhabitants/km^2^, although these values are highly irregular with most people concentrated in towns and villages^36, 37^. The level of landscape fragmentation by infrastructures, such as roads, railways or settlements, shows a contrasting gradient—rather remote and homogeneous mountain ranges are surrounded by intensively used valleys and basins with high human population densities^38^. In the Kysuce site, permanent presence and long-term reproduction of Eurasian lynx, grey wolf (*Canis lupus*) and brown bear (*Ursus arctos*) was recorded, while in the Javorníky site, only lynx and wolf reproduction was documented. In the Beskydy site, only lynx reproduction was confirmed during the study period^32^, author´s unpublished data.

### Camera-trapping

Camera-trapping was conducted throughout an 80-day winter period (November–February) and during five consecutive seasons (2015, 2016, 2017, 2018, 2019—the year means the beginning of the camera-trapping period lasting to the next year) in all study sites. The length of the camera-trapping survey was set according to the recommendations of Weingarth et al.^39^. Each period was divided into 16 trapping occasions of 5 days each^8, 9, 39^. The study sites encompassed by the outermost cameras was estimated using the minimum convex polygon (MCP^40^) and ranged from 811.10 to 918.49 km^2^ in the Beskydy site, from 223.79 to 273.35 km^2^ in the Javorníky site and from 320.60 to 417.88 km^2^ in the Kysuce site (Fig. 1). To ensure that all animals had a non-zero capture probability^41^, we placed cameras systematically to avoid any gap larger than the smallest home range of a female lynx in the Carpathians and set at least two cameras per female home range^3^. The smallest published home range size for female lynx is 124 km^2^ in the Carpathians^42^; therefore its radius (6.30 km) was used as the maximum spacing between cameras. The availability of suitable cameras (n = 16 to 60; Table 1) resulted in the average distance to the nearest neighbouring cameras (Point distance tool in ArcMap 10.7.1^43^) from 2.08 km (standard deviation, hereafter SD, ± 1.18) to 2.37 km (± 0.95 km) in Beskydy, from 1.24 km (± 0.94) to 2.28 km (± 1.04) in Javorníky and from 1.81 km (± 1.33) to 3.29 km (± 0.69) in the Kysuce site (Fig. 1, Table S1). Thefts of camera traps in the beginning of trapping sessions caused the maximum spacing to be higher than 6.3 km in two cases during a 5-year period (Beskydy 2017 and Kysuce 2016). One camera with white flash or infrared camera (Cuddeback Ambush, Cuddeback C123, Cuddeback H20 IR, Cuddeback Green Bay, USA; Browning Spec Ops Advantage, Browning Morgan, USA) was installed at each camera-trapping site. Selection of camera sites with the highest probability of lynx detection was based on our previous knowledge obtained by snow tracking and opportunistic camera-trapping (game trails, marking sites and rocky ridges^32, 44, 45^).

**Table 1:** Basic parameters of the systematic lynx camera-trapping in five consecutive seasons within three study sites in the Western Carpathians.

Study site	Season	Survey length (days)	Unique captures	Independent lynx (M/F/LF/ND) + J	Trap days (total/effective)	Cameras/Lynx detections
Beskydy	2015	80	19	5 (2/1/2/0) 3	3680/3455	46/10
	2016	80	33	6 (3/1/2/0) 3	4480/3845	56/17
	2017	80	11	5 (3/1/1/0) 2	4800/4370	60/7
	2018	80	18	3 (2/0/1/0) 1	4160/3855	52/10
	2019	80	40	7 (5/0/2/0) 6	3920/3625	49/16
Javorníky	2015	80	58	5 (3/1/1/0) 3	2000/1855	25/16
	2016	80	62	5 (1/2/2/0) 3	2720/2410	34/22
	2017	80	51	6 (4/1/1/0) 3	3120/2800	39/17
	2018	80	101	7 (4/3/0/0) 0	4240/3915	53/39
	2019	80	101	6 (4/1/1/0) 3	3680/3365	46/36
Kysuce	2015	80	37	7 (4/1/2/0) 4	1280/1270	16/13
	2016	80	22	7 (4/0/1/2) 2	2240/1980	28/13
	2017	80	65	12 (8/2/1/1) 2	3120/2785	39/25
	2018	80	58	9 (6/1/2/0) 4	3200/2680	40/23
	2019	80	61	12 (7/1/0/4) 0	2720/2525	34/18

*M* male, *F* female, *LF* leading female, *ND* not determined, *J* juvenile.

### Identification of individuals and determination of social status

Reliable identification of captured animals (see Fig. S2—Photographic database of independent lynx) was ensured by using a detailed photo-database of lynx individuals collected during the opportunistic camera-trapping (from 2009 in Beskydy and Javorníky, from 2013 in Kysuce) conducted throughout the year as well as by using data from previous deterministic surveys in all study sites^32, 44–46^. Multiple photos were obtained, especially at marking sites, allowing us to assign both body flanks to one individual. Individuals were identified by comparison of coat patterns, particularly on the hind limbs, fore limbs and flanks^8, 11^. At least three well-trained observers in each site were involved in the intensive identification process of identifying lynx individuals by using an online multipurpose photographic database and cross-check verification. Identification of individuals and data processing followed minimum camera-trapping standards reported by Choo et al.^47^.

Sex and age category of individuals was determined from clearly visible genital parts and captures of leading females with kittens in the pictures, as well as from videos gained through previous deterministic and opportunistic camera-trapping^32, 44–46^ or through genetic analyses^29^. Lynx individuals detected during the five seasons of deterministic camera-trapping were divided according to their social status into three different categories: adult (A)—individual older than two years that was present for at least 12 months in the study site (territorial lynx)^29^; subadult (S)—independent individual in the second year of life with well-known life history (known mother and birth year); not determined status (ND)—all other individuals with unknown or not determined status (Table S2).

### Spatially explicit capture-recapture model

Only independent lynx individuals > 1 year old (adults and subadults) were integrated into analyses^9^. Multiple captures of the same individual at a particular trap site, during the same trapping occasion, were considered as a single capture^8^. The capture of kittens of a known leading female was considered as a capture of that female^48^. Lynx densities were estimated by means of spatially explicit capture-recapture analyses (SCR). For SCR analyses, we used the software SPACECAP version 1.1.0^49, 50^ implemented within R software v. 3.6.0^51^. To meet the used model key assumptions^6, 9^, we used trap response present, spatial capture-recapture model half-normal detection and Bernoulli´s encounter process with the same parameter values applied (Markov chains with 80 000 iterations, a burn-in period 40 000, thinning rate 3 and data augmentation 100) as in Kubala et al.^11^. The assumption of demographic population closure was tested through CloseTest^52, 53^. CloseTest suggested population closure in 8 out of 15 seasons (Table S4). Since results could be potentially influenced by the fact that 3 individuals moved between sites within one season, we also calculated a scenario where only captures matching the site of the first capture in that season were retained (Table S6). These changes had no significant effect on total estimates of population density so we present results where population density is estimated independently for each site with all individuals.

To find the minimum buffer width for which density estimates stabilize, we created a series of state-spaces with buffers ranging from 2 to 24 km (with increment of 2 km) around the MCP surrounding all camera traps^11^. The state-space was described as a grid of 576–1999 equally spaced potential home range centres (1.5 × 1.5) resulting in state-space sizes ranging between 1269 and 4497.25 km^2^ (Table 1 and Fig. 1). Lynx densities were estimated per 100 km^2^ of suitable habitat. Proportions of suitable and unsuitable habitat were derived from CORINE Land Cover 2012^54^, where all different types of forests, shrubs and natural grasslands were considered as suitable habitat for lynx, following Kubala et al.^11^. Chain convergence was tested using Gelman-Rubin's test^55^ where values below 1.1 indicate convergence^56^. Finally, estimates of lynx density obtained in all study sites were compared between each pair of consecutive seasons using the calculation of the coefficient of variation and fold changes. The Kruskal–Wallis (KW) test was used to test differences in density estimates among study sites and seasons. The Spearman's rank correlation coefficient (SRCC) was used to test trends in average annual densities over the five seasons. The calculations were conducted in R^51^.

### The multistate closed robust design

The multistate closed robust design models were run in MARK^57^ and estimated three parameters per site: (i) apparent survival rate (φ), which is the probability of surviving and staying in a sample site; (ii) transition probability (ψ), which represents the probability of moving from one site to another; and, (iii) capture probability (*P*). The modelling approach assumes that no site transitions occurred within a primary period, i.e. season^58, 59^. However, we acknowledge that 2.2% of the captures violated this assumption. One adjustment was made to minimize this violation, using the approach of Chabanne et al.^60^. If an individual was captured in two different sites within a primary period, we retained captures matching the site of the first capture recorded in that primary period. We also analysed the dataset where the captures matching site of the second captures were retained and the results were similar (same survival rate and the best model selected); thus we present only the first option here. Models were ranked using the Akaike information criterion (AICc^61^). The model with most support by AICc (highest AICc weight) was selected as the most parsimonious model.

### Estimation of individual’s turnover

The individual’s turnover was calculated as the proportion of individuals that were recorded during a monitoring survey in the previous season but were not recorded in a consecutive season. The individual’s turnover between consecutive seasons was calculated for different sexes and age categories (all individuals vs. adults). If an individual was captured in two different sites within the same season, the calculation of turnover rate included this particular individual only in the site where it was captured for the first time (the same way as it was done in multi-state closed robust design dataset).

### Ethics approval

The research used non-invasive methods only. The entry to protected areas was approved by Trenčín District Office (No. OU-TN-OSZP1-2014/49/3475) and by the agreement with the State Nature Conservancy of the Slovak Republic (No. ŠOP SR/12/2018).

## Results

### Camera-trapping survey

In total, we identified 53 independent lynx within 737 unique captures obtained during 44 735 effective trap days from all sites and seasons. Sex was identified for 47 individuals (29 males, 18 females), while 6 individuals remained undetermined. The age was successfully identified in 33 individuals, of which 28 were adults and 5 were subadults. For 13 individuals, we were not able to determine their social status. The status of these individuals did not change during the survey. Additionally, the age category changed for 6 individuals from subadult to adult and for one individual from undetermined to adult (Table 1, Table S2). Camera-trapping efficiency ranged from 83.8 to 99.2% among all sites and seasons. Five individuals were recorded in two different study sites—four in Beskydy and Javorníky, and one in Kysuce and Javorníky. Moreover, three out of these five individuals were recorded in two different sites within the same camera-trapping season (Table S2). Altogether, 93 pictures and videos of lynx were excluded from analyses due to their insufficient quality for lynx determination (see minimum reporting standards in Table S3).

### Estimates of population density

Density estimates decreased rapidly with increasing buffer width and began to stabilize at buffer size ≥ 8 km. Stabilisation among all study sites and seasons occurred mostly in buffer size 10 and 12 km (Table S1). The posterior mean baseline encounter rate (λ_0_) (posterior SD) varied from 0.02 (± 0.01) to 0.22 (± 0.06) and the posterior movement parameter varied from 3.17 (± 0.69) to 9.83 (± 0.44) km among all sites and seasons (Table 2).

**Table 2:** Population size and density estimates of Eurasian lynx during five seasons of systematic camera-trapping in three study sites in the Western Carpathians.

Study site	Season	Suitable habitat	Posterior density	Population size	Encounter rate	Movement parameter σ	Bayesian *p* value
Beskydy	2015	1527.75	0.50 ± 0.15	7.71 ± 2.31	0.035 ± 0.014	5.87 ± 1.33	0.68
	2016	2322	0.37 ± 0.11	8.78 ± 2.60	0.029 ± 0.008	9.83 ± 2.44	0.76
	2017	1239.75	1.08 ± 0.58	13.49 ± 7.29	0.020 ± 0.013	4.39 ± 2.94	0.59
	2018	1350	0.26 ± 0.07	3.63 ± 0.99	0.043 ± 0.018	7.80 ± 0.83	0.69
	2019	1908	0.49 ± 0.10	9.48 ± 2.07	0.060 ± 0.017	7.04 ± 1.04	0.79
Javorníky	2015	1017	0.61 ± 0.14	6.21 ± 1.48	0.152 ± 0.036	5.40 ± 0.87	0.62
	2016	1188	0.59 ± 0.16	7.02 ± 1.95	0.116 ± 0.024	5.55 ± 0.85	0.64
	2017	859.5	0.93 ± 0.22	8.06 ± 1.89	0.068 ± 0.016	4.75 ± 0.71	0.74
	2018	1048.5	0.93 ± 0.21	9.85 ± 2.24	0.069 ± 0.010	4.92 ± 0.52	0.88
	2019	900	0.90 ± 0.20	8.12 ± 1.84	0.210 ± 0.032	3.73 ± 0.32	0.88
Kysuce	2015	1062	0.97 ± 0.24	10.30 ± 2.61	0.227 ± 0.069	4.23 ± 0.64	0.49
	2016	1005.75	1.38 ± 0.40	11.71 ± 3.44	0.093 ± 0.036	3.17 ± 0.69	0.66
	2017	1156.5	1.61 ± 0.30	18.68 ± 3.50	0.101 ± 0.020	3.95 ± 0.41	0.73
	2018	990	1.26 ± 0.23	12.52 ± 2.35	0.131 ± 0.026	3.89 ± 0.37	0.69
	2019	994.5	1.85 ± 0.35	18.45 ± 3.56	0.109 ± 0.024	3.54 ± 0.42	0.59

**Figure 2:**
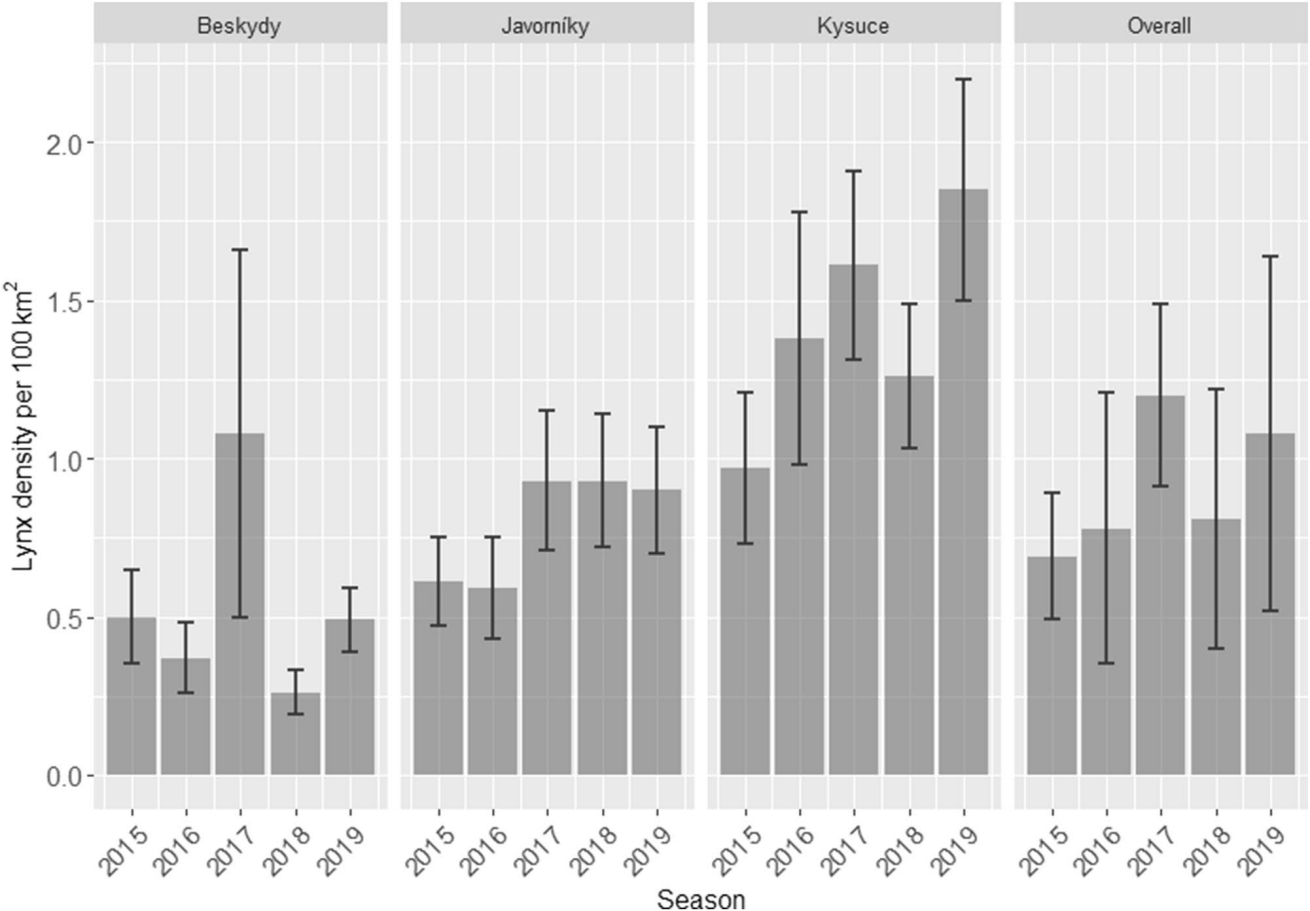
Estimates of Eurasian lynx density obtained by systematic camera-trapping during five consecutive seasons in three study sites (posterior mean ± SD) and average values for the whole region (average ± SD) in the Western Carpathians.

Overall, mean posterior densities varied between 0.26 (± 0.07) and 1.85 (± 0.35) independent lynx/100 km^2^ suitable habitat. In particular, posterior densities ranged between 0.26 (± 0.07) and 1.08 (± 1.58) in Beskydy (mean 0.54 lynx/100 km^2^), between 0.59 (± 0.16) and 1.19 (± 0.27) in Javorníky (mean 0.79 lynx/100 km^2^) and between 0.97 (± 0.24) and 1.85 (± 0.35) independent lynx/100 km^2^ suitable habitat in the Kysuce site (mean 1.41 lynx/100 km^2^) (Fig. 2, Table 2). Over the period of this study we recorded a 4.1-fold change in lynx density in Beskydy, a 1.9-fold change in Kysuce and a 1.5-fold change in Javorníky (Fig. 2). The coefficient of variation (CV) was the highest (58.7%) in Beskydy, followed by the Kysuce (23.9%) and the Javorníky sites (22.2%). The average annual density estimates calculated for all three sites together ranged from 0.69 to 1.20 lynx/100 km^2^ with no significant increase over the five seasons (SRCC, R_S_ = 6, *p* = 0.23). Density estimates varied significantly between all study sites (KW test, χ^2^ = 9.63, *p* = 0.008) but not between seasons (KW test, χ^2^ = 2.16, *p* = 0.7). Bayesian *P* values suggesting model adequacy ranged from 0.49 to 0.88 among all sites and seasons. Gelman-Rubin diagnostics indicated convergence for all models. Values of all estimated parameters were below 1.1, except for season 2017 in Beskydy.

### Estimates of apparent survival and transition probability

The best fitting model according to the AICc weight was that of constant apparent survival, constant transition rate and capture probability varied by site and season [*P*(site × season)]. The difference from the models where the transition rate and apparent survival varied by site or sex were not particularly high (ΔAICc ˂ 2.2), thus suggesting those models as having good support as the best one^61^. The best competing models are listed in Table 3, however not all model combinations converged and we were therefore limited in the number of models available.

**Table 3:** Comparison of seven competing models built on apparent survival (φ), transition rate ψ, probability of capture (*P*) and abundance (N) ranked from the best candidate model (lowest AICc value).

Models	AIC_c_	ΔAIC_c_	AIC_c_ weight	Model likelihood	Parameters	Deviance
A φ (.) ψ (.) *P* (site*p) N (site*p)	1916.9	0	0.31207	1	17	1881.5629
B φ (site) ψ (.) *P* (site*p) N (site*p)	1917.6	0.8	0.2104	0.6742	19	1878.0336
C φ (.) ψ (site) *P* (site*p) N (site*p)	1918.1	1.2	0.17098	0.5479	22	1871.9034
D φ (site) ψ (site) *P* (site*p) N (site*p)	1920	2.1	0.10747	0.3444	24	1868.4221
E φ (sex) ψ (sex) *P* (site*p) N(site*p)	1919.1	2.2	0.10261	0.3288	20	1877.2973
F φ (sex) ψ (.) *P* (site*p) N ( site*p)	1920.5	3.6	0.05107	0.1637	19	1880.8652
G φ (sex) ψ (sex) *P* (site*p) N (site*p*sex)	1921.5	4.6	0.03102	0.0994	21	1877.5084

Parameters were constant (.) or varied by site, sex or season—primary period (*p*). Probability of capture was equal to recapture (*P* = c).

The overall apparent survival rate was 0.63 ± 0.055 and overall transition rate 0.03 ± 0.019 according to the best model. While not significant, estimates of apparent survival rate when varying by sex was higher for males (0.67 ± 0.072) than females (0.6 ± 0.087). Lynxes with undetermined sex had the lowest survival (0.47). Survival rate also varied (non-significantly) among sites with higher apparent survival rate estimated in Beskydy (0.70 ± 0.102) and Javorníky (0.74 ± 0.092) than in Kysuce (0.52 ± 0.085). Transition rate of males was 0.05 ± 0.029 between season, in contrast to none for females (˂0.001). Transition rate of undetermined sex, however, was much higher (0.54 ± 0.00). Capture probability was significantly higher in Javorníky (0.54 ± 0.02) than in Beskydy (0.24 ± 0.02) and Kysuce (0.24 ± 0.016) (Figure S1).

### Turnover and persistence of individuals

**Figure 3:**
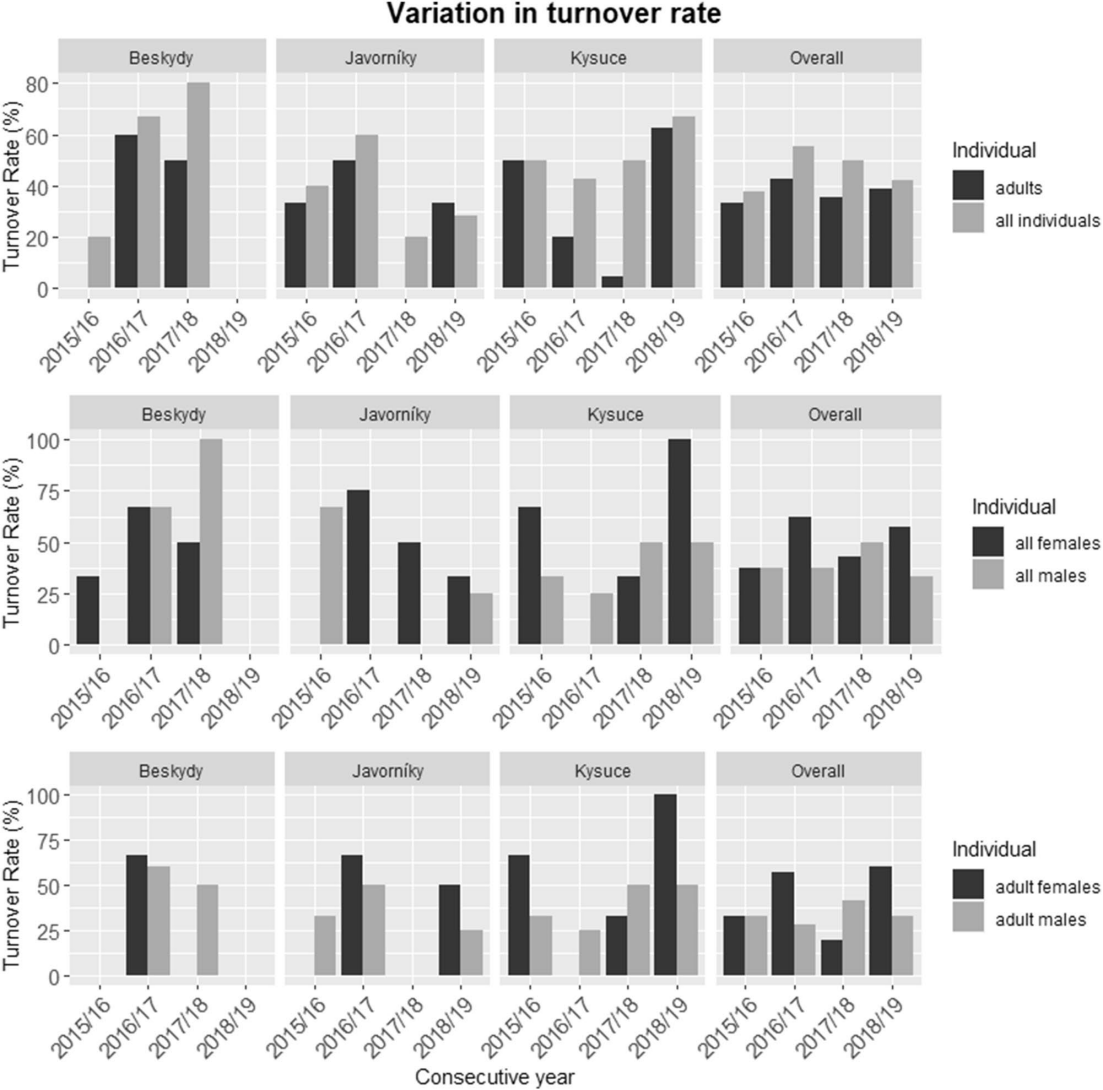
Variation in turnover rates of different categories (adults vs all individuals; all females vs all males; adult females vs adult males) among all sites during four consecutive seasons of systematic camera-trapping.

In total, the average turnover was 46.3 ± 8.06% including all independent lynx (n = 53) and 37.6 ± 4.22% for adults (n = 33). The overall turnover among all sites and seasons varied from 33.3 to 50% in males and from 37.5 to 62.5% in females. In adult males and adult females, the total average turnover reached 34.2 ± 5.44% and 42.6 ± 19.2%, respectively (Fig. 3, Table S5). Only three individuals were captured during all seasons and nine individuals in ≥ 3 seasons (Table S2).

## Discussion

### Density estimates

More accurate estimation of several population parameters has become possible thanks to the currently widespread use of camera traps and recent developments in spatial models^63, 64^. Our overall lynx density range obtained within this study (0.26–1.85 lynx/100 km^2^ suitable habitat) corresponds to spatial lynx density estimates reported from other areas in Europe e.g., French Jura and Vosges Mountains (0.24–0.91 lynx/100 km^2^)^13^ or Swiss Alps (1.04–1.47 lynx/100 km^2^)^9, 48^. Moreover, the mean posterior density of 0.26 lynx/100 km^2^ from the Beskydy site is the second lowest spatial density reported from Europe just higher than the Doubs (0.24 lynx/100 km^2^)^13^.

The average density values obtained in three study sites (Beskydy 0.54 lynx/100 km^2^, Javorníky 0.79 lynx/100 km^2^, Kysuce 1.41 lynx/100 km^2^) are in accordance with the centre-periphery hypothesis as well as the “abundant centre” distribution hypothesis, which assume that species reach their highest abundance in the centre of their range and decline in abundance toward the range edges^26^. This hypothesis is also supported by the density values reported in the previous studies from the Slovak Carpathians. In particular, lynx density from the western edge (Beskydy) was similar to density reported in the Štiavnica Mts (0.58 lynx /100 km^2^ suitable habitat)^11^, situated at the southern periphery of lynx distribution in Slovakia. The density reported from the Javorníky site was similar to the density obtained in the adjacent Strážov Mts (0.97 ± 0.25 lynx/100 km^2^ suitable habitat)^20^. The density estimated for the Kysuce site was comparable to the density values from the Muránska planina NP or the Vepor Mts (1.47 ± 0.37 and 1.20 ± 0.49 lynx/100 km^2^ suitable habitat, respectively), situated in the central part of Slovakia^18, 19^. However, our results confirmed relatively high density fluctuations in all three study sites and, thus, local densities in the central part of Slovakia may also significantly fluctuate among seasons. For example, the one-season density estimation recently reported in Velká Fatra Mts (0.81 ± 0.29 lynx/100 km^2^ suitable habitat)^11,^ also situated in the centre, might represent the estimation at the lower bounds.

Non spatial and spatial capture-recapture modelling approaches have been developed and used for density estimation of populations. Similarly to Avgan et al.^65^, we have omitted using conventional non-spatial CR models and only the SCR model was used to estimate lynx densities. This model seems to be more reliable for lynx density estimation in comparison to standard closed CR models^13, 48^. Besides the model used for density estimation, there are also several other factors that might affect density estimates and make the comparison between studies disputable.

First, density estimation can be influenced by the length of the camera-trapping survey and the season in which it is conducted. We conducted our deterministic survey during an 80-day period, although the majority of studies from the Carpathians and other European populations used 60-day length e.g.^9, 11, 13, 18^. The extended period length in our study was set in order to obtain a sufficient number of captures and re-captures of individuals (the most crucial factor in obtaining a reliable and robust density estimation) mostly in the Beskydy, the site with the lowest density values situated at the periphery. Moreover, the additional test of demographic closure supported the 80-day period rather than 60-day (Table S4) and a longer camera-trapping survey is highly recommended to obtain sufficient data for reliable estimates of demographic parameters^39, 66^. Although we used an extended period length, we conducted our survey outside the mating season and dispersal period to avoid violating the demographic closure. However, in the 2017 season in Beskydy we detected no convergence in chains and a relatively high level of standard deviation of posterior density estimates. Results obtained in this particular season could be affected by several factors, such as several malfunctions of cameras, low recapture rates of individuals^67^ or different movement patterns among sex and social categories of lynx^9^, which might decrease detection probability.

### Demographic changes

Substantial interannual density fluctuation and fold changes (1.5–4.1-fold change) of the native Carpathian populations recorded within our study supported previous findings of fluctuated densities observed on reintroduced lynx populations in Western Europe. Comparable fold changes (up to threefold change) of lynx densities were observed in the North Western Alps^24^, Swiss Jura Mts.^68^ and French Jura and Vosges Mts.^13^. However, previous long-term density estimates in Jura Mts., based mainly on telemetry research, report a fairly constant trend^69^. Generally, similar density fluctuations using long-term camera-trapping surveys were also reported also other territorial felids, e.g. tigers^21, 22^ and jaguars^66^. In contrast, no substantial fluctuation with relatively stable trends was recorded for cheetah^70^.

The overall apparent survival, which consists of true survival and permanent emigration, was 63% in all sites. The advantage of the multi-state closed robust design approach is to estimate transition rate between sites. Our transition rate among years and any site was about 3% per year, which indicates a small but consistent connection between populations of lynx from each site. No camera trap study estimated the apparent survival of Eurasian lynx, therefore limiting our comparison with only a few older studies based on radiotelemetry. Survival reached 63% in North-Eastern Poland^71^, 76% for adults and 53% for subadults in Swiss Jura^72^ and it varied by sex and age category in three study sites in Scandinavia^73^: survival rates ranged within 77–83% for adult males, 85–86% for adult females, 57–74% for subadult males and 43–90% for subadult females. Although our camera-trapping study did not allow for the estimation of apparent survival for adults and subadults categories separately due to limited history of all individuals, it seems our overall survival rates are among the lowest reported in Europe, also taking into account the low transition rate. Females particularly did not move among sites indicating strong female philopatry and male biased dispersal, as was also documented by the genetic analyses in our area^29^ and in Finland^74^.

We found high individual’s turnover (average for all independent lynx 46.3%, and for adults 37.6%) and low persistence of adults over the five consecutive seasons (3 out of 29 individuals). These long-term findings agree with the occasional high individual´s turnover (up to 80%) and low persistency (mean 12.7 months) of lynx individuals previously documented by pilot surveys in Štiavnica Mts. and Veľká Fatra NP^11^. Similarly, the high individual’s turnover was also documented in the Javorníky and Beskydy sites during our previous extensive camera-trapping survey^45^ and also by non-invasive genetic sampling conducted in this area^29^. Low individual persistence and low age of captured residents were reported during radio-tracking research in the Jura Mts.^72^ and most recently in the Northern Hessian subpopulation in Germany^75^. A high individual turnover rate (up to 89%) in combination with low persistence was reported for other felids, e.g. Geoffroy’s cats^76^ and tigers^23^.

Fluctuating densities, relatively low apparent survival and high turnover rates could be affected by several ecological (e.g. food and habitat availability, diseases, competition) and human-induced (e.g. poaching, road mortality, habitat fragmentation) factors. Here we discuss the most relevant hypotheses for observed demographic changes, starting with the least plausible one.

In a human dominated landscape, lynx distribution is shaped by a trade-off between the availability of preferred prey and the amount of human activity^77–79^. Population numbers of wild ungulates, especially roe deer as the most selected prey of lynx in Europe^80^, are at historical maximums in Slovakia and the Czech Republic^81, 82^. Therefore, it is unlikely that the observed fluctuations of lynx density have been driven by the lack of natural prey. Moreover, roe deer is more abundant in Beskydy and Javorníky than in Kysuce, while lynx density was lower in those two sites compared to the Kysuce site^83^. Similarly, the proportion of suitable habitat for lynx and the level of human activities are comparable among all study sites and lynx do not use all suitable habitats, especially in the Beskydy site^29^. Additionally, we did not observe any signs of a disease outbreak in this area. Therefore, we believe in a limited influence of the ecological factors mentioned above on asynchronous density fluctuation and high turnover rates in our study sites.

Despite the fact that a percentage of animals die naturally (diseases, intraspecific killing, aging, etc.)^84, 85^, we assume that a high proportion of adult mortality might be caused by anthropogenic factors as reported in other regions, e.g. Scandinavia^73^, Alps^72^ or Dinaric Mts^86^. We found a low survival rate and relatively high turnover of both sexes, especially adult females. This might rather indicate the influence of artificial phenomena, such as anthropogenic caused mortality, e.g. poaching^87, 88^ or road mortality^84^. Several cases of lynx poaching (n = 5), collisions with vehicle/train (n = 5) or orphaned kittens (n = 2) were documented by chance in our study sites from 2002 to 2020 (authors’ unpublished data). High anthropogenic pressure (significant level of human-induced mortality) was also documented in other areas of the Western Carpathians^11, 89^ and Europe^90^. Moreover 10% of Czech hunters surveyed in the study by^88^ admitted that they personally killed lynx. Occasional dips in survival caused by human-induced mortality are likely to cause drops in recruitment in subsequent years, depressing the population size as observed in Beskydy and Kysuce between seasons 2017—2018 (Fig. 2). Subsequent rebound of survival due to habitat and prey availability may result in an increased population size in following years, as seen in Beskydy and Kysuce in season 2019. Although the average annual density in our study sites over the five consecutive seasons showed a slightly increasing trend (Fig. 2), we have not observed lynx expansion westwards into other surrounding areas within the lynx historical range^91^. Only occasional lynx dispersals have been documented in the Moravian region over the last decade^32^. This underlines a poor dispersing ability of lynxes, especially females^92, 93^. On the other hand, human interventions might also play a significant role in limiting population expansion^29, 94^.

Contrary to our expectations, apparent survival rate was not higher in the core (Kysuce) and females did not have overall higher survival. Differences were not significant, but the opposite trend could be partly explained by lower capture probabilities in Kysuce than in Javorníky (Fig. S1). Another reason could be that higher population density causes higher intra-specific competition, which is reflected as depressed apparent survival rates^23^. More lynxes leads to higher encounter rates with hunters who perceive lynx negatively, which could also increase the social conflict and probability of illegal killing. However, these factors need more detailed investigation. In many species, demographic traits do not follow centre-peripheral hypothesis and local ecological effects may be more influential than the position of population within the range^25^. In other words, geographically peripheral populations are not necessarily ecologically marginal.

### Conclusions for management and conservation

Average annual density estimates for the whole region (all three sites together) varied between 0.69 and 1.20 lynx/100 km^2^ suitable habitat and showed substantial variation in lynx density over the five seasons of systematic camera-trapping. Based on average annual densities obtained within this study, and using 28 090 km^2^ of suitable lynx habitat occupied by lynx in Slovakia according to Kubala et al.^11^, we estimate lynx population size in Slovakia to vary between 193 and 337 individuals. The lowest value (season 2015) is very similar to 197 individuals estimated by Kubala et al.^11^ in 2014/15 and may represent the population minimum. Estimates from season 2017 and 2019 (337 and 303 individuals, respectively) reached a similar level as the most recent estimate (280 individuals) reported by Kubala et al.^20^. This indicates that lynx population numbers varied within slightly lower values than those officially reported by the State Nature Conservancy for European Commission during 2013–2018 (300–400 individuals)^95^.

A multi-seasonal camera-trapping survey conducted in three study sites situated at the centre-periphery gradient enabled the first robust density estimation for lynx in the Western Carpathians. Since the density estimates varied greatly between consecutive seasons, our study demonstrates that long-term camera-trapping surveys might be needed not only for evaluation of population trends but for reliable estimates of population size as well. Special attention should be paid to the native populations, because these may serve as a source of individuals for repatriation and reinforcement purposes in the near future. Moreover, the fluctuating densities, relatively low apparent survival and high turnover rates presented in this study (and others e.g.^11, 29^) indicate that the West Carpathian population is facing several human-induced factors, which might negatively influence the otherwise favourable conservation status of this population. In order to maintain a favourable population status, we call for more rigorous investigation of illegal killing and for its reduction by establishing a network of wildlife forensic experts, by strengthening scene investigation and by prosecuting illegal activities through law enforcement. Poaching, as well as habitat loss from landscape fragmentation and an increasing number of lynx-vehicle collisions, seem to be the most limiting factors restricting population growth and dispersion of lynx in the human-dominated landscapes across Europe^86, 87^.

## Supplementary information

Supplementary Information.

## Data Availability

The datasets analysed during the current study are not publicly available due to sensitivity of the occurrence data of endangered species but are available from the corresponding author on reasonable request.
