# Supplementary material for: Multi-seasonal systematic camera-trapping reveals fluctuating densities and high turnover rates of Carpathian lynx on the western edge of its native range
Source: Sci Rep. 2021 Apr 29;11:9236. doi: 10.1038/s41598-021-88348-8 (PMC8085240; doi:10.1038/s41598-021-88348-8)
Supplement: Supplementary file 1 — Supplementary Information. [file 41598_2021_88348_MOESM1_ESM.docx]

**Multi-seasonal systematic camera-trapping reveals fluctuating densities and high turnover rates of Carpathian lynx on the western edge of its native range**

Martin Duľa, Michal Bojda, Delphine B. H. Chabanne, Peter Drengubiak, Ľuboslav Hrdý, Jarmila Krojerová-Prokešová, Jakub Kubala, Jiří Labuda, Leona Marčáková, Teresa Oliveira, Peter Smolko, Martin Váňa & Miroslav Kutal

**Supplementary material**

*Table S1: Mean minimum distance (MMD) between camera-trapping sites (± SD), buffer width stabilisation of densities and percentage proportion of suitable habitat centres from state space in three sites and all seasons of deterministic camera-trapping*

| **Site** | **Season** | **MCP**  **(km^2^)** | **MMD***  **(km)** | **Buffer**  **width (km)** | **Proportion of**  **suitable habitat**  **(%)** | **State space**  **(km^2^)** |
| --- | --- | --- | --- | --- | --- | --- |
| Beskydy | 2015 | 870.84 | 2.28 ± 1.21 | 12 | 55.43 | 2756.25 |
|  | 2016 | 843.41 | 2.08 ± 1.18 | 20 | 51.63 | 4497.25 |
|  | 2017 | 918.49 | 2.13 ± 1.25 | 8 | 58 | 2137.5 |
|  | 2018 | 811.1 | 2.09 ± 1.17 | 10 | 57.86 | 2333.25 |
|  | 2019 | 852.26 | 2.37 ± 0.95 | 16 | 52.31 | 3647.25 |
| Javorníky | 2015 | 223.79 | 2.28 ± 1.04 | 12 | 65.89 | 1543.5 |
|  | 2016 | 238.48 | 1.65 ± 1.12 | 14 | 64.63 | 1838.25 |
|  | 2017 | 268.72 | 1.47 ± 1.13 | 10 | 66.32 | 1296 |
|  | 2018 | 270.8 | 1.24 ± 0.94 | 12 | 65.26 | 1606.5 |
|  | 2019 | 273.35 | 1.33 ± 1.12 | 10 | 67.11 | 1341 |
| Kysuce | 2015 | 320.6 | 3.29 ± 0.69 | 12 | 64.04 | 1658.25 |
|  | 2016 | 412.6 | 2.83 ± 1.15 | 8 | 65.16 | 1269 |
|  | 2017 | 417.88 | 1.99 ± 1.25 | 12 | 62.23 | 1858.5 |
|  | 2018 | 411.91 | 1.81 ± 1.33 | 10 | 64.52 | 1534.5 |
|  | 2019 | 411.03 | 2.13 ± 1.23 | 10 | 64.43 | 1534.5 |

*Table S2: Lynx independents recorded during five season of deterministic camera-trapping in three areas. A – adult, S –subadult, ND – not determined. S/A – individual captured first season as subadult*

| **Area** | **Individual** | **Sex** | **Status** | **First detection** | **2015** | **2016** | **2017** | **2018** | **2019** |
| --- | --- | --- | --- | --- | --- | --- | --- | --- | --- |
| Beskydy | Boris1 | ♂ | S | Nov-19 |  |  |  |  | ● |
|  | Bronislav | ♂ | A | Sep-18 |  |  |  | ● | ● |
|  | Čestmír | ♂ | ND | Jan-20 |  |  |  |  | ● |
|  | Draža | ♀ | A | Dec-10 | ● | ● | ● | ● | ● |
|  | František | ♂ | A | Sep-16 |  |  |  | ● | ● |
|  | Jíří | ♂ | S/A | Sep-14 | ● | ● | ● |  |  |
|  | Kamila | ♀ | S | Sep-14 | ● |  |  |  |  |
|  | Kornélie | ♀ | S/A | Dec-16 |  |  | ● |  | ● |
|  | Lenka | ♀ | A | Mar-11 |  | ● |  |  |  |
|  | Licous | ♂ | A | Mar-10 | ● | ● |  |  |  |
|  | Olda2 | ♂ | A | Dec-19 |  |  |  |  | ● |
|  | Otmar | ♂ | ND | Nov-17 |  |  | ● |  |  |
|  | Rufus3 | ♂ | S | Nov-16 |  |  | ● |  |  |
|  | Žigmund5 | ♂ | ND | Nov-16 |  | ● |  |  |  |
|  | Žofka | ♀ | A | Aug-15 | ● | ● |  |  |  |
| Javorníky | Boris1 | ♂ | S | Nov-19 |  |  |  |  | ● |
|  | Gabi | ♀ | S/A | Nov-17 |  |  |  | ● | ● |
|  | Heřmína | ♀ | A | Mar-12 | ● | ● | ● | ● | ● |
|  | Hortenzia | ♀ | S/A | Jan-15 | ● | ● |  |  |  |
|  | Jolana | ♀ | ND | Sep-16 |  | ● |  |  |  |
|  | Karla | ♀ | S | Nov-16 |  |  | ● |  |  |
|  | Král | ♂ | A | Mar-09 | ● |  |  |  |  |
|  | Ľubo4 | ♂ | ND | Nov-15 | ● |  |  |  |  |
|  | Lucka | ♀ | A | Sep-16 |  | ● |  | ● |  |
|  | Olda2 | ♂ | A | Oct-10 | ● | ● | ● | ● |  |
|  | Rufus3 | ♂ | S/A | Dec-17 |  |  | ● | ● | ● |
|  | Štěpán | ♂ | AR | Oct-16 |  |  | ● | ● | ● |
|  | Žigmund5 | ♂ | A | Dec-16 |  |  | ● | ● | ● |
| Kysuce | Danka | ♀ | A | Oct-12 | ● | ● | ● |  |  |
|  | Duch | ♂ | A | Sep-13 | ● |  |  |  |  |
|  | Emanuel | ♂ | ND | Mar-19 |  |  |  |  | ● |
|  | Hubert | ♂ | ND | Nov-19 |  |  |  |  | ● |
|  | Job | ♂ | A | Jan-17 |  | ● | ● |  |  |
|  | Johan | ♂ | A | Sep-17 |  |  | ● | ● |  |
|  | Ľubo4 | ♂ | ND | Oct-15 | ● |  |  |  |  |
|  | Ľudmila | ♀ | A | Aug-17 |  |  | ● | ● |  |
|  | Marcela | ♀ | ND | Oct-18 |  |  |  | ● |  |
|  | Martin | ♂ | A | Nov-15 | ● | ● | ● | ● | ● |
|  | Maťo | ♂ | A | Aug-17 |  |  | ● | ● |  |
|  | Michal | ♂ | A | Sep-16 |  |  | ● | ● |  |
|  | Monika | ♀ | S | Jul-18 |  |  |  |  | ● |
|  | Naďa | ♀ | A | Jun-15 | ● |  |  |  |  |
|  | No name 1 | NA | ND | Nov-16 |  | ● |  |  |  |
|  | No name 2 | NA | S/A | Jan-18 |  |  | ● |  | ● |
|  | No name 3 | ♂ | ND | Oct-19 |  |  |  |  | ● |
|  | No name 4 | NA | ND | Sep-19 |  |  |  |  | ● |
|  | No name 5 | NA | ND | Nov-19 |  |  |  |  | ● |
|  | No name 6 | NA | ND | Dec-19 |  |  |  |  | ● |
|  | No name 7 | NA | S | Oct-15 |  | ● |  |  |  |
|  | Ondrinka | ♀ | A | Jul-16 |  |  | ● | ● |  |
|  | Ondro | ♂ | A | May-18 |  |  |  | ● | ● |
|  | Peter | ♂ | ND | Nov-19 |  |  |  |  | ● |
|  | Rozeťáčik | ♂ | A | Sep-17 |  |  | ● |  |  |
|  | Stijn | ♂ | A | Oct-16 |  | ● | ● |  |  |
|  | Tichomír | ♂ | A | Oct-18 |  |  |  | ● | ● |
|  | Vlado | ♂ | A | Feb-17 |  |  | ● |  |  |
|  | Vratko | ♂ | A | Nov-14 | ● | ● |  |  |  |
|  | Zoja | ♀ | A | Sep-13 | ● |  |  |  |  |

*Table S3: Minimum reporting standards for our study design according to the Choo et al. (2020).*

| **Site** | **Season** | **Number of photographs/videos obtained^1^** | **Number of individuals with complete identification** | **Number of partial identities** | **Number of identified captures** | **Number of unique captures^2^** | **Number of unidentified captures^3^** | **Number of discrepancies found** |
| --- | --- | --- | --- | --- | --- | --- | --- | --- |
| **Beskydy** | **2015** | 130 | 5 | 0 | 127 | 19 | 3 | 0 |
|  | **2016** | 195 | 6 | 0 | 194 | 33 | 1 | 0 |
|  | **2017** | 101 | 5 | 0 | 100 | 11 | 1 | 0 |
|  | **2018** | 42 | 3 | 0 | 42 | 18 | 0 | 0 |
|  | **2019** | 243 | 7 | 0 | 243 | 40 | 0 | 1 |
| **Javorníky** | **2015** | 99 | 5 | 0 | 96 | 58 | 3 | 0 |
|  | **2016** | 215 | 5 | 0 | 198 | 62 | 17 | 0 |
|  | **2017** | 98 | 5 | 0 | 84 | 51 | 14 | 0 |
|  | **2018** | 208 | 7 | 0 | 197 | 101 | 11 | 0 |
|  | **2019** | 172 | 6 | 0 | 160 | 101 | 12 | 0 |
| **Kysuce** | **2015** | 85 | 7 | 0 | 84 | 37 | 1 | 0 |
|  | **2016** | 35 | 6 | 1 | 34 | 22 | 0 | 1 |
|  | **2017** | 98 | 12 | 0 | 95 | 65 | 3 | 0 |
|  | **2018** | 116 | 9 | 0 | 98 | 58 | 18 | 0 |
|  | **2019** | 125 | 10 | 2 | 116 | 61 | 9 | 0 |

*^1^all photo and videos including multiple captures;* *^2^ number of unique captures used for SCR analyses; ^3^all captures which were excluded from analyses due to insufficient quality for lynx determination*

*Table S4: Results of population closure tests by CloseTest (Stanley & Richards 2004) for the 80-day (16 occasions) and the 60-day period (12 occasions) for all sites and seasons*

| **Site** | **Season** | **16 occasions** | **12 occasions** |
| --- | --- | --- | --- |
| Beskydy | 2015 | 0.007 | 0.238 |
|  | 2016 | 0 | 0.114 |
|  | 2017 | 0.799 | -1 |
|  | 2018 | 0 | 0 |
|  | 2019 | 0.136 | 0 |
| Javorníky | 2015 | 0.898 | 0.0064 |
|  | 2016 | 0.155 | 0 |
|  | 2017 | 0.674 | 0.998 |
|  | 2018 | 0.99 | 0.866 |
|  | 2019 | 0 | 0.002 |
| Kysuce | 2015 | 0 | 0.007 |
|  | 2016 | 0.009 | 0.026 |
|  | 2017 | 0 | 0 |
|  | 2018 | 0.228 | 0.353 |
|  | 2019 | 0.614 | 0.002 |

*Table S5: Percentage rate of individual turnover in all consecutive seasons of deterministic camera-trapping (information provided in brackets represent the number of individuals that were recorded during a monitoring survey in the previous season but were not recorded in a consecutive season and the total number of individuals from which the turnover was calculated)*

| **Category** | **Site** | **2015/16** | **2016/17** | **2017/18** | **2018/19** |
| --- | --- | --- | --- | --- | --- |
| All individuals | Beskydy | 20% (1/5) | 66.7% (4/6) | 80% (4/5) | 0% (0/3) |
|  | Javorníky | 40% (2/5) | 60% (3/5) | 20% (1/5) | 28.6% (2/7) |
|  | Kysuce | 50.0% (3/6) | 42.9% (3/7) | 50% (6/12) | 66.7% (6/9) |
|  | Overall | 37.5% (6/16) | 55.6% (10/18) | 50.0% (11/22) | 42.1% (8/19) |
| All males | Beskydy | 0% (0/2) | 66.7% (2/3) | 100% (3/3) | 0% (0/2) |
|  | Javorníky | 66.7% (2/3) | 0% (0/1) | 0% (0/3) | 25% (1/4) |
|  | Kysuce | 33.3% (1/3) | 25% (1/4) | 50% (4/8) | 50% (3/6) |
|  | Overall | 37.5% (3/8) | 37.5% (3/8) | 50 % (7/14) | 33.3% (4/12) |
| All females | Beskydy | 33.33% (1/3) | 66.7% (2/3) | 50% (1/2) | 0% (0/1) |
|  | Javorníky | 0% (0/2) | 75% (3/4) | 50% (1/2) | 33.3% (1/3) |
|  | Kysuce | 66.7% (2/3) | 0% (0/1) | 33.3% (1/3) | 100% (3/3) |
|  | Overall | 37.5% (3/8) | 62.5% (5/8) | 42.9% (3/7) | 57.1% (4/7) |
| Adults | Beskydy | 0% (0/3) | 60% (3/5) | 50% (1/2) | 0% (0/3) |
|  | Javorníky | 33.3% (1/3) | 50% (2/4) | 0% (0/4) | 33.3% (2/6) |
|  | Kysuce | 50% (3/6) | 20% (1/5) | 45.5% (5/11) | 62.5% (5/8) |
|  | Overall | 33.3% (4/12) | 42.9% (6/14) | 35.3% (6/17) | 38.9% (7/18) |
| Adult males | Beskydy | 0% (0/1) | 50% (1/2) | 100% (1/1) | 0% (0/2) |
|  | Javorníky | 50% (1/2) | 0% (0/1) | 0% (0/3) | 25% (1/4) |
|  | Kysuce | 33.3% (1/3) | 25% (1/4) | 50% (4/8) | 50 % (3/6) |
|  | Overall | 33.3% (2/6) | 28.58% (2/7) | 41.67% (5/12) | 33.3% (4/12) |
| Adult females | Beskydy | 0% (0/2) | 66.7% (2/3) | 0% (0/1) | 0% (0/1) |
|  | Javorníky | 0% (0/1) | 66.7% (2/3) | 0% (0/1) | 50% (1/2) |
|  | Kysuce | 66.6% (2/3) | 0% (0/1) | 33.3% (1/3) | 100% (2/2) |
|  | Overall | 33.3% (2/6) | 57.2% (4/7) | 20% (1/5) | 60% (3/5) |

*Table S6: Population size and density estimates in a scenario where captures of 3 lynx detected in two sites within one season were retained only at sites of the first capture (see Methods and compare with Table 2): –Lynx Ľubo removed from Kysuce* ***2015^1^****, lynx Rufus removed from Javorníky* ***2017^2^****, lynx Boris removed from Javorníky* ***2019^3^***

| **Site** | **Season** | **Suitable** | **Posterior** | **Population** | **Encounter** | **Movement** | **Bayesian** |
| --- | --- | --- | --- | --- | --- | --- | --- |
|  |  | **habitat** | **Density** | **size** | **rate** | **parameter σ** | **p value** |
| Beskydy | 2015 | 1527.75 | 0.50 ± 0.15 | 7.71 ± 2.31 | 0.035 ± 0.014 | 5.87 ± 1.33 | 0.68 |
|  | 2016 | 2322 | 0.37 ± 0.11 | 8.78 ± 2.60 | 0.029 ± 0.008 | 9.83 ± 2.44 | 0.76 |
|  | 2017 | 1239.75 | 1.08 ± 0.58 | 13.49 ± 7.29 | 0.020 ± 0.013 | 4.39 ± 2.94 | 0.59 |
|  | 2018 | 1350 | 0.26 ± 0.07 | 3.63 ± 0.99 | 0.043 ± 0.018 | 7.80 ± 0.83 | 0.69 |
|  | 2019 | 1908 | 0.49 ± 0.10 | 9.48 ± 2.07 | 0.060 ± 0.017 | 7.04 ± 1.04 | 0.79 |
| Javorníky | 2015 | 1017 | 0.61 ± 0.14 | 6.21 ± 1.48 | 0.152 ± 0.036 | 5.40 ± 0.87 | 0.62 |
|  | 2017 | 859.5 | 0.93 ± 0.22 | 8.06 ± 1.89 | 0.068 ± 0.016 | 4.75 ± 0.71 | 0.74 |
|  | **2017^2^** | **1041.75** | **0.74 ± 0.22** | **7.78 ± 2.35** | **0.076 ± 0.018** | **4.77 ± 0.76** | **0.67** |
|  | 2018 | 1048.5 | 0.93 ± 0.21 | 9.85± 2.24 | 0.069 ± 0.010 | 4.92 ± 0.52 | 0.88 |
|  | **2019^3^** | **1341** | **0.74 ± 0.17** | **6.71 ± 1.61** | **0.209 ± 0.033** | **3.79 ± 0.36** | **0.90** |
| Kysuce | **2015^1^** | **915.75** | **0.87 ± 0.21** | **8.04 ± 1.95** | **0.233 ± 0.074** | **4.06 ± 0.61** | **0.52** |
|  | 2016 | 1005.75 | 1.38 ± 0.40 | 11.71 ± 3.44 | 0.093 ± 0.036 | 3.17 ± 0.69 | 0.66 |
|  | 2017 | 1156.5 | 1.61 ± 0.30 | 18.68 ± 3.50 | 0.101 ± 0.020 | 3.95 ± 0.41 | 0.73 |
|  | 2018 | 990 | 1.26 ± 0.23 | 12.52 ± 2.35 | 0.131 ± 0.026 | 3.89 ± 0.37 | 0.69 |
|  | 2019 | 994.5 | 1.85 ± 0.35 | 18.45 ± 3.56 | 0.109 ± 0.024 | 3.54 ± 0.42 | 0.59 |

Figure S1: Capture probabilities of lynx individuals for all sites and seasons. Estimates from the best model A (Table 3) with higher and lower 95% Confidence Intervals.


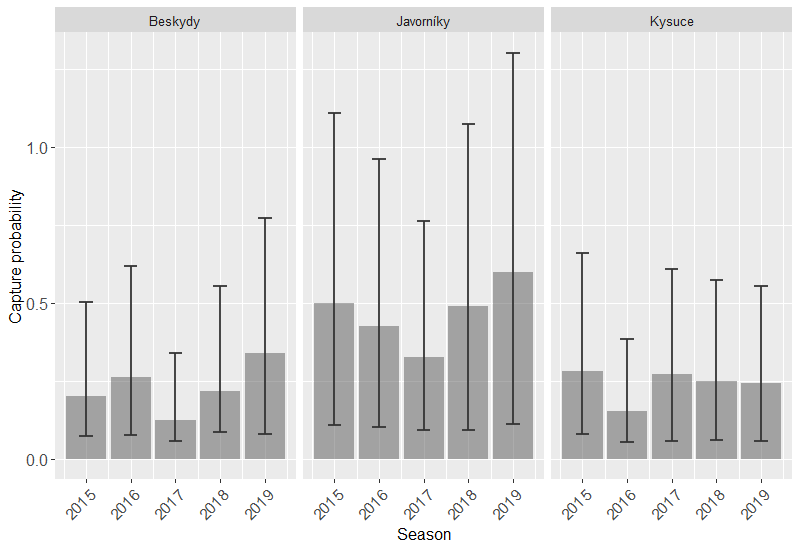


*Figure S2: Photographic database of independent lynxes. Name/Sex/Status/First detection (A– adult, S –subadult, ND – not determined. S/A – individual captured first season as subadult)*

BESKYDY


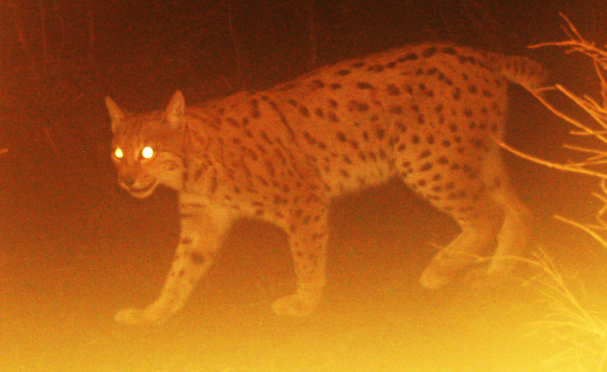

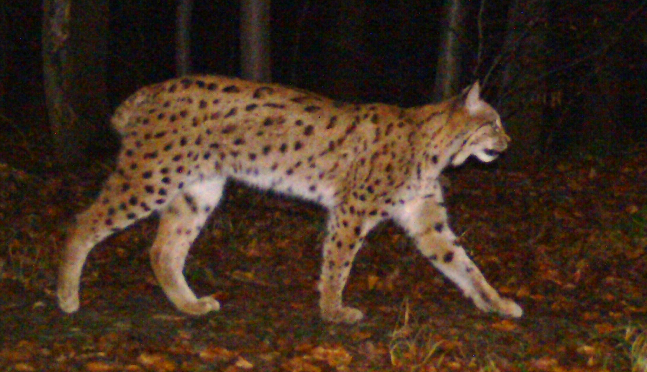
Boris, ♂, S, Nov-19


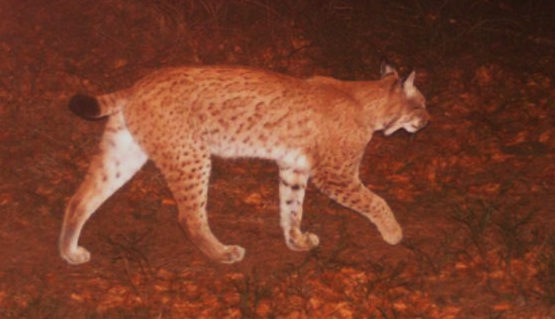

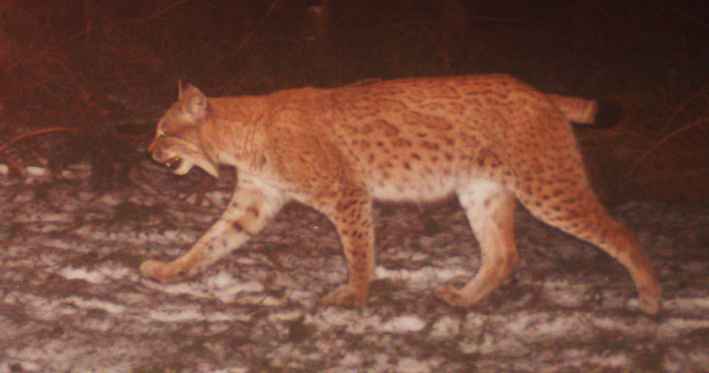
Bronislav, ♂, A, Sep-18


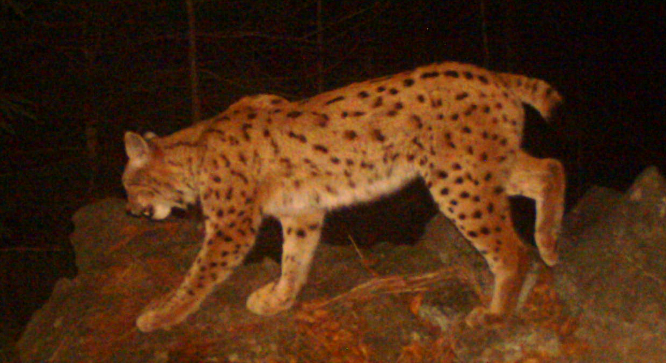

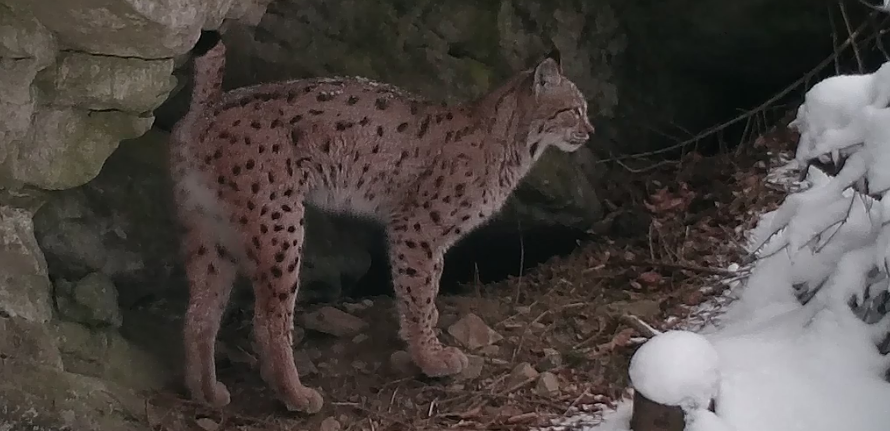
Čestmír, ♂, ND, Jan-20


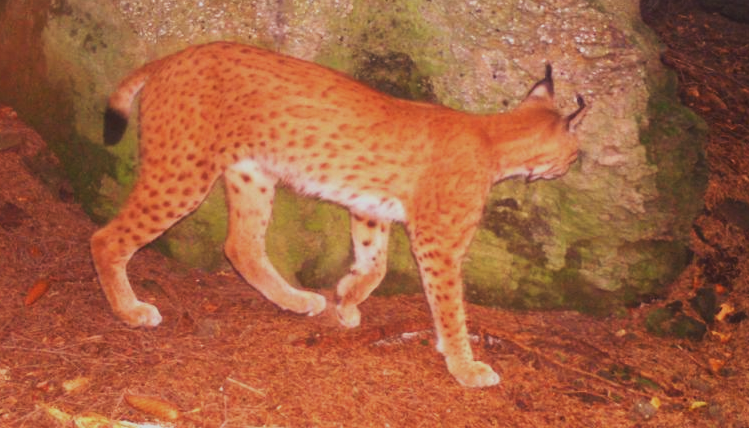
Draža, ♀, A, Dec-10


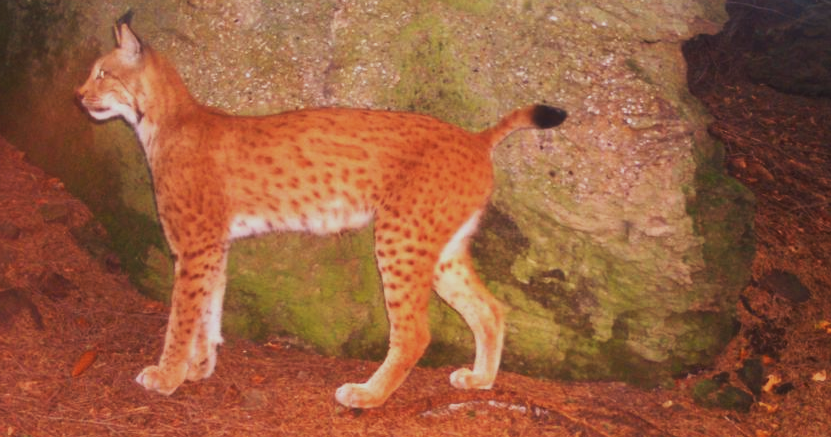


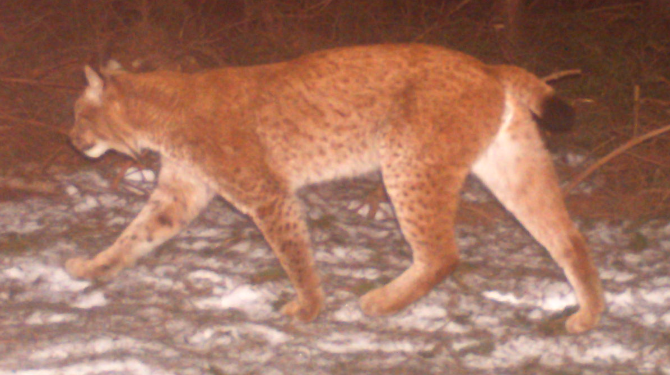

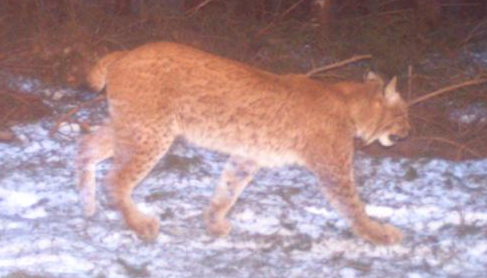
František, ♂, A, Sep-16


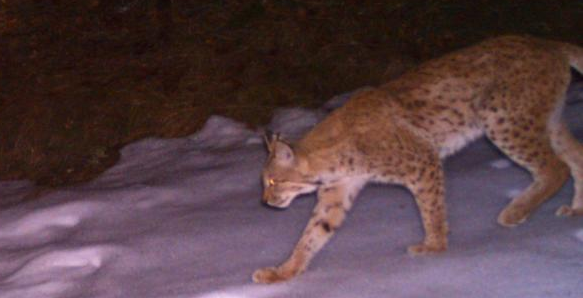

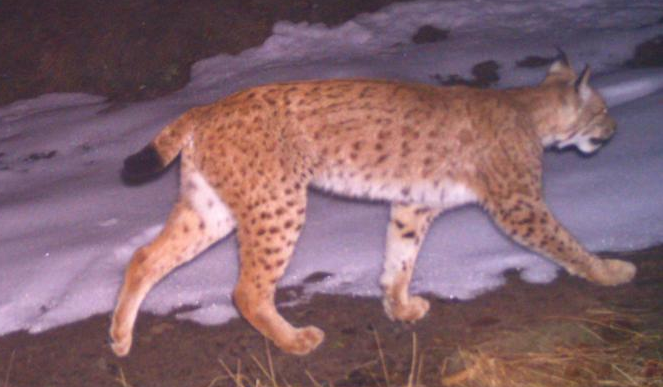
Kamila, ♀, S, Sep-14


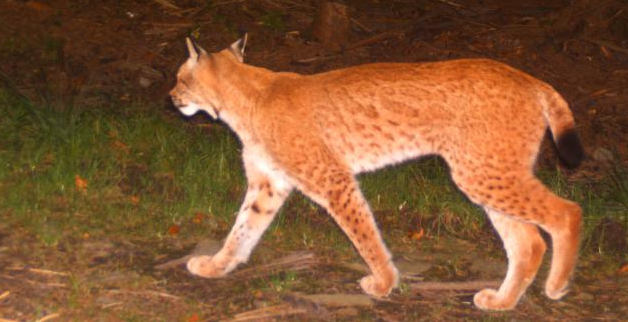

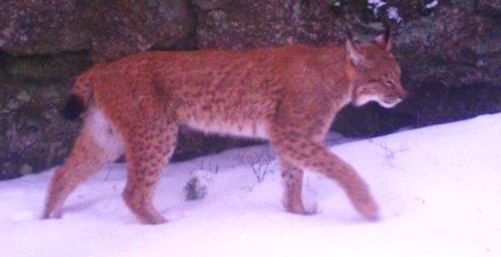
Kornélie, ♀, S/A, Dec-16


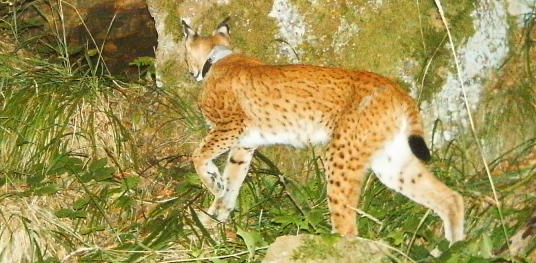

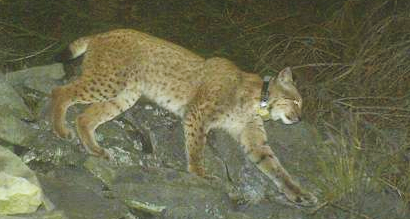
Lenka, ♀, A, Mar-11


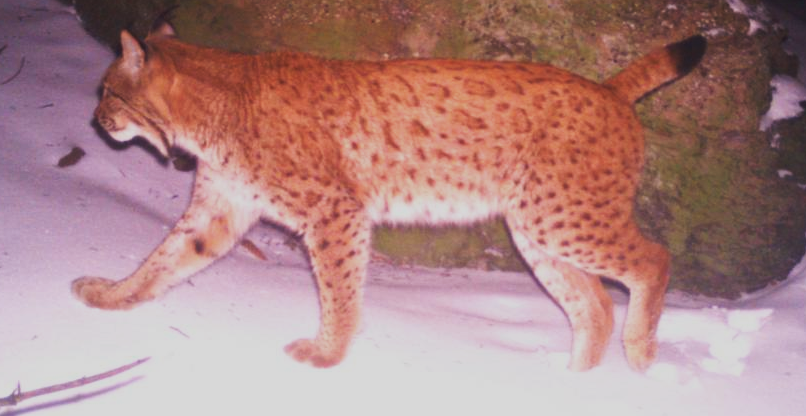

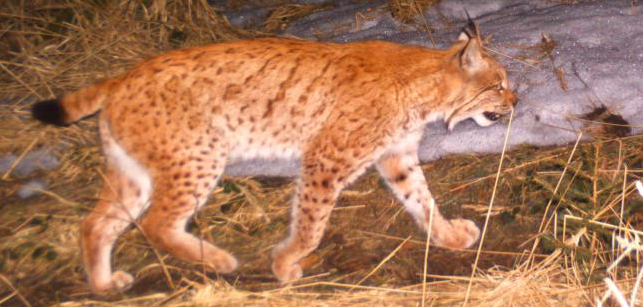
Licous, ♂, *A, Mar-10*


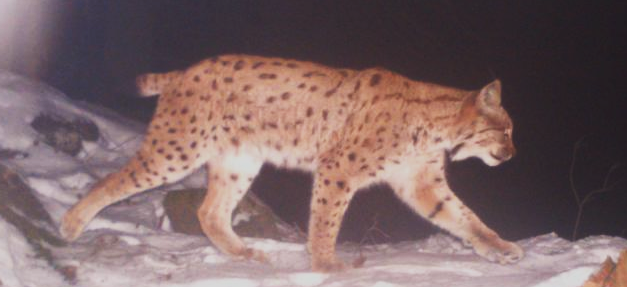

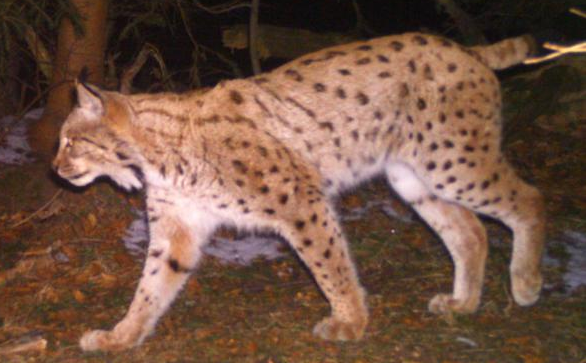
Olda, ♂, A, Dec-19


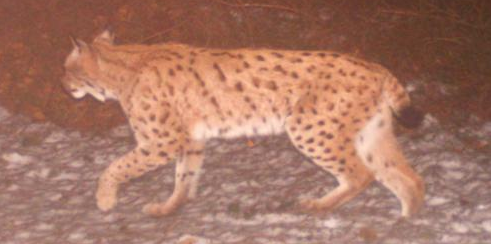

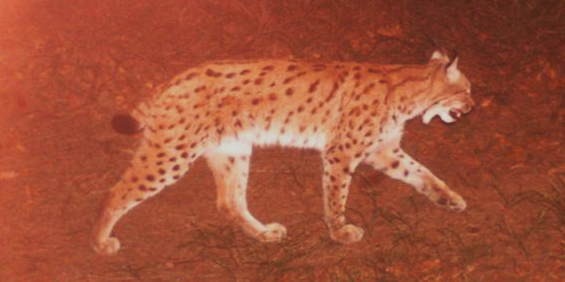
Otmar, ♂, ND, Nov-17


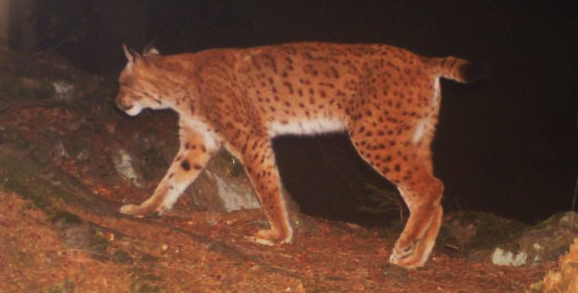

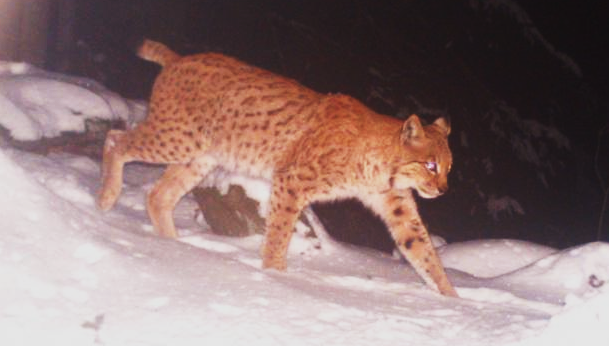
Rufus, ♂, S, Nov-16


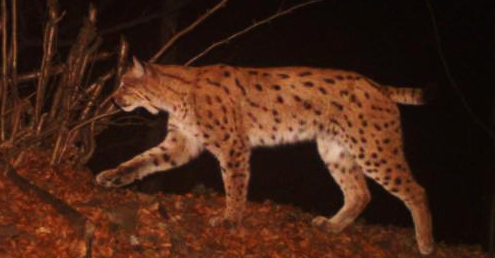

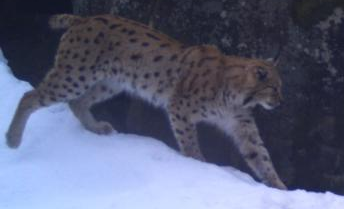
Žigmund, ♂*,* ND, Nov-16


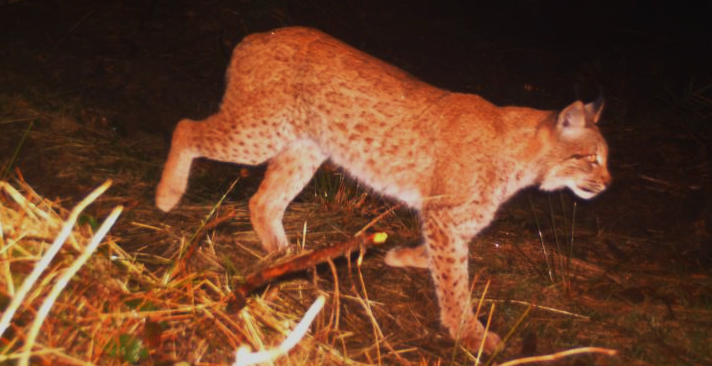

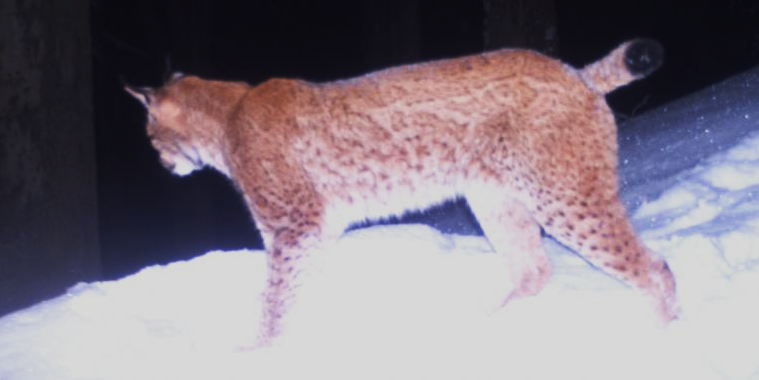


Žofka*,* ♀*, A, Aug-15*

JAVORNÍKY


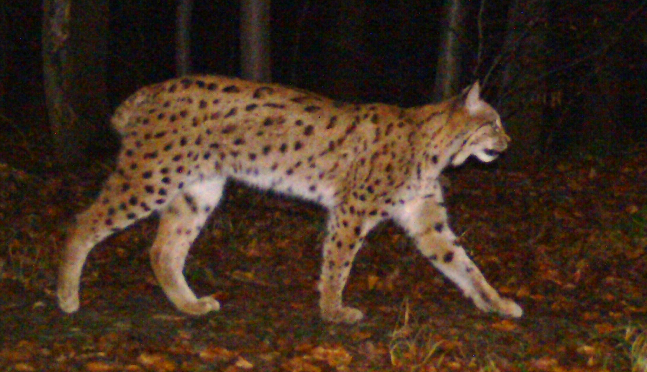

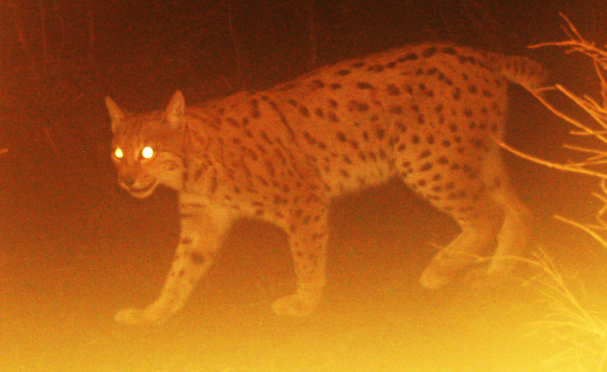
Boris, ♂, S, Nov-19


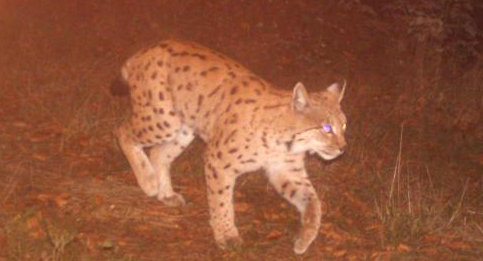

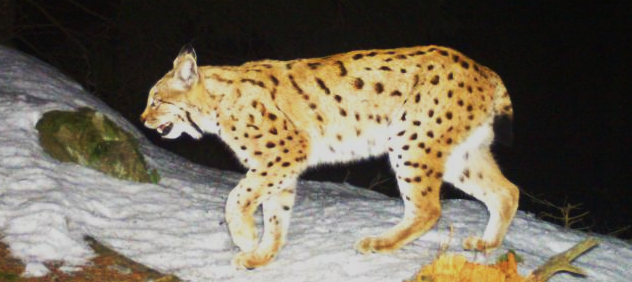
Gabi, ♀*,* S/A, Nov-17


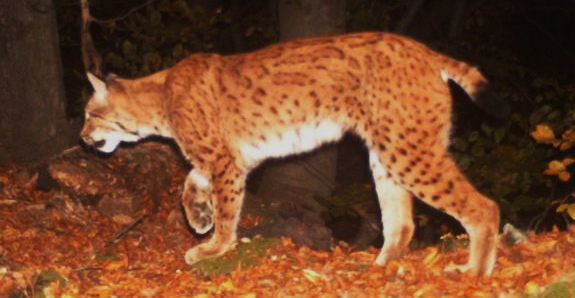

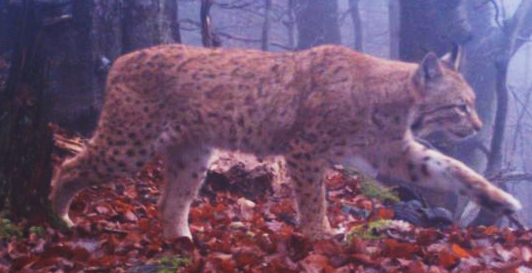
Heřmína, ♀, A, Mar-12


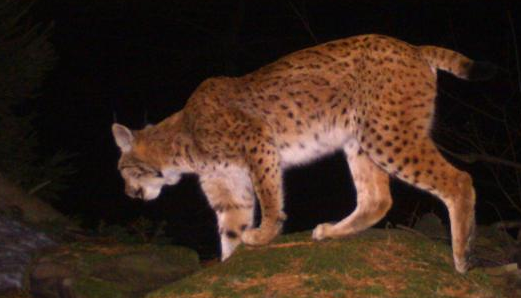

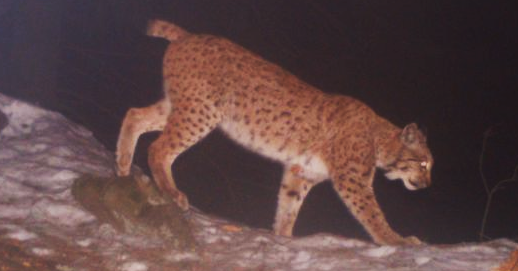
Hortenzia, ♀, S/A, Jan-A5


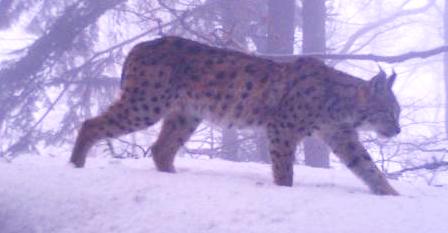

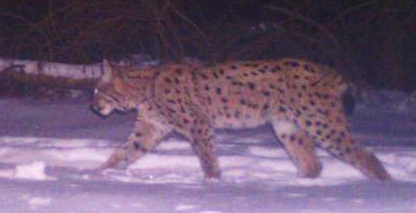
Jolana, ♀, ND, Sep-16


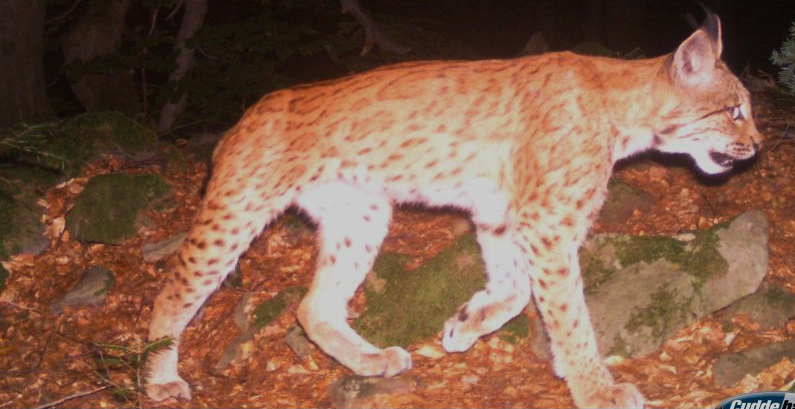

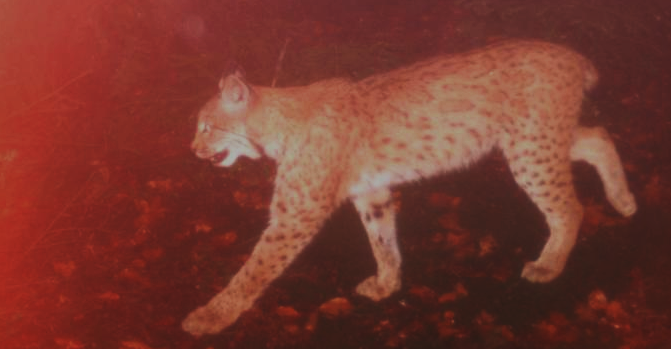
Karla, ♀, S, Nov-16


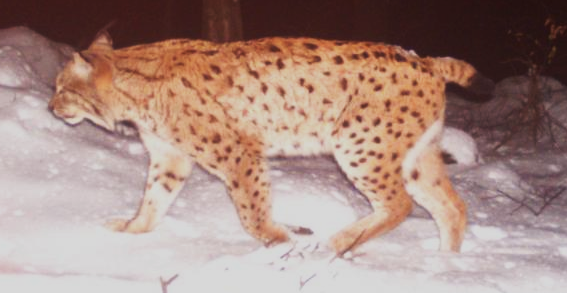

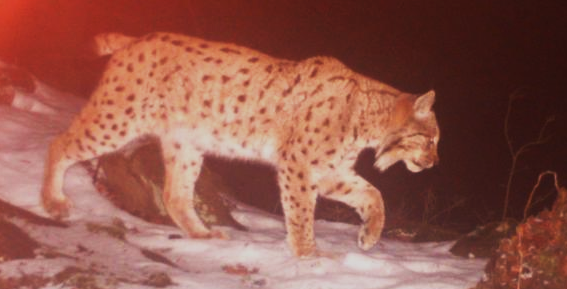
Král, ♂, A, Mar-09


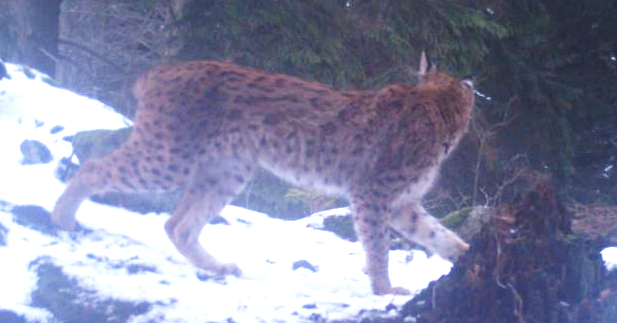

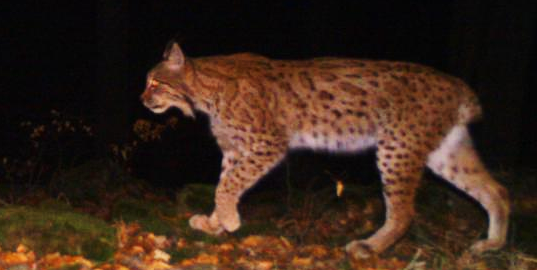
Ľubo, ♂, F, Nov-15


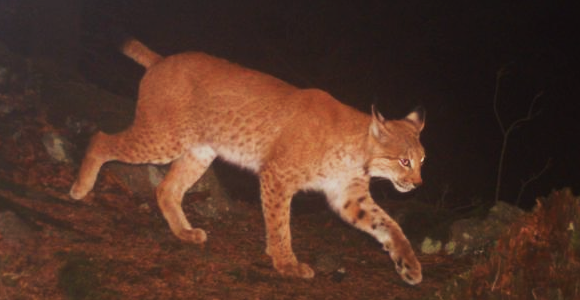

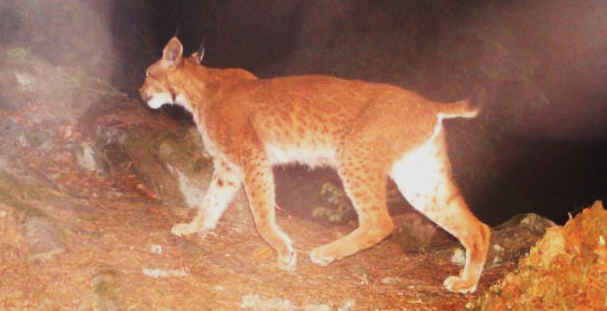
Lucka*,* ♀*, A, Sep-16*


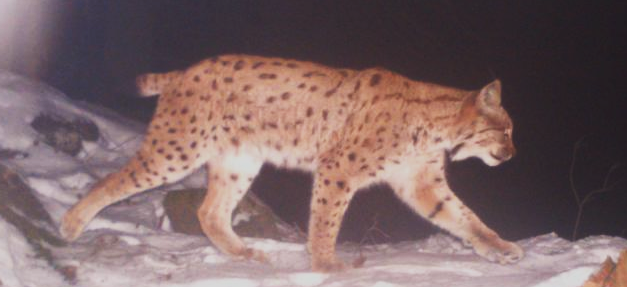

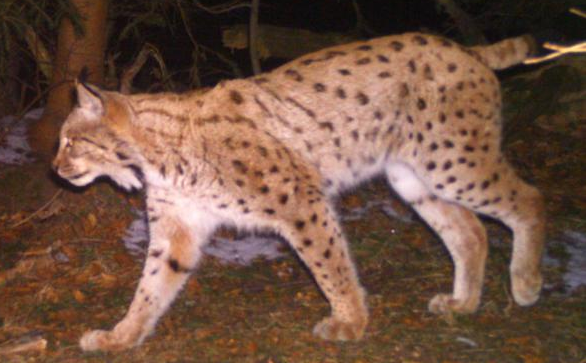
Olda, ♂, A, Oct-10


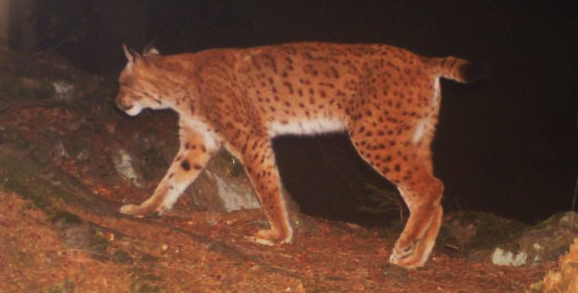

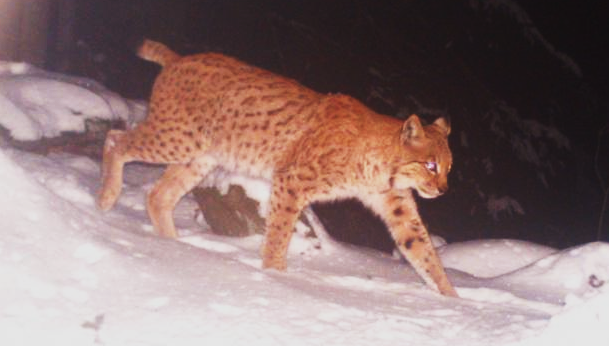
Rufus, ♂, S, Nov-16


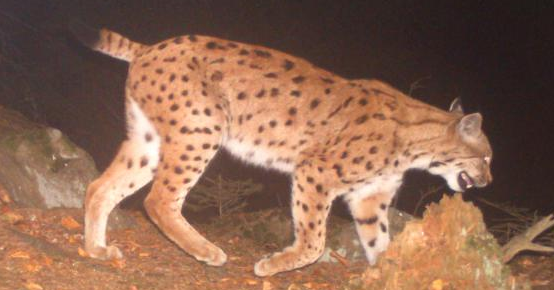

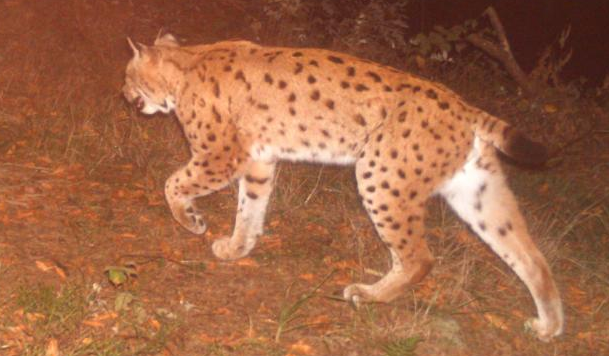
Štěpán, ♂, A, Oct-16


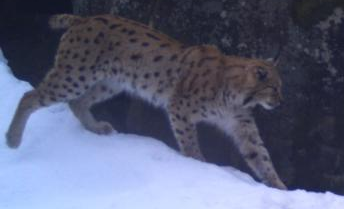

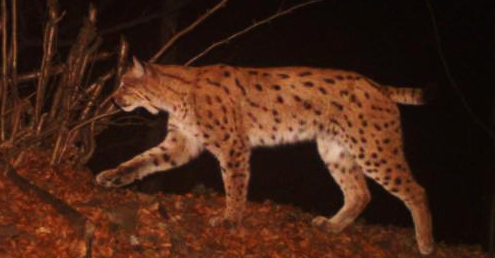
Žigmund, ♂*,* F, Nov-16

KYSUCE


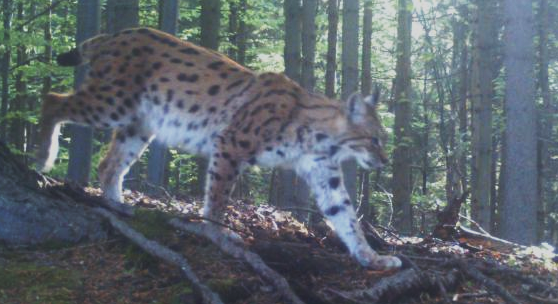

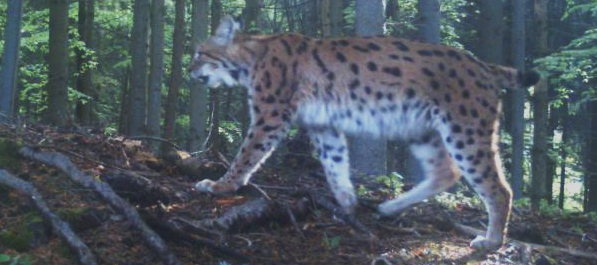
Danka, ♀, *A, Oct-12*


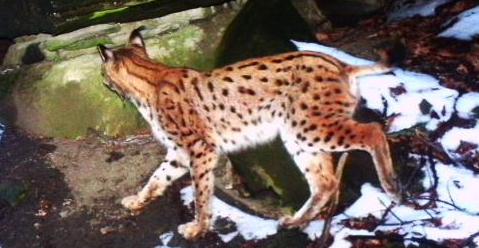

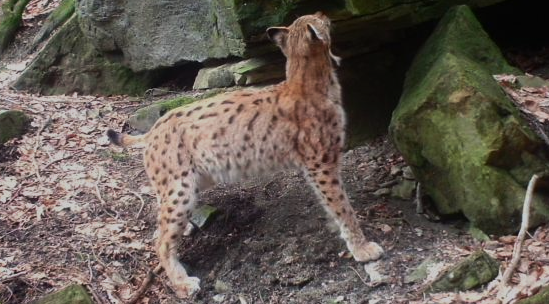
Duch, ♂, A, Sep-13


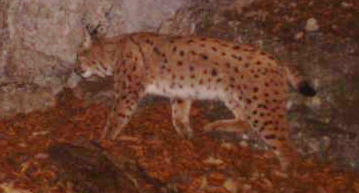
Emanuel, ♂, ND, Mar-19


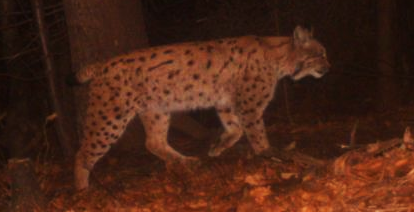


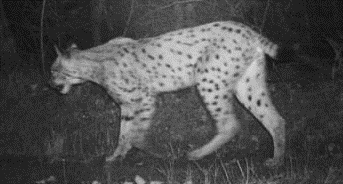
Hubert, ♂, ND, Nov-19


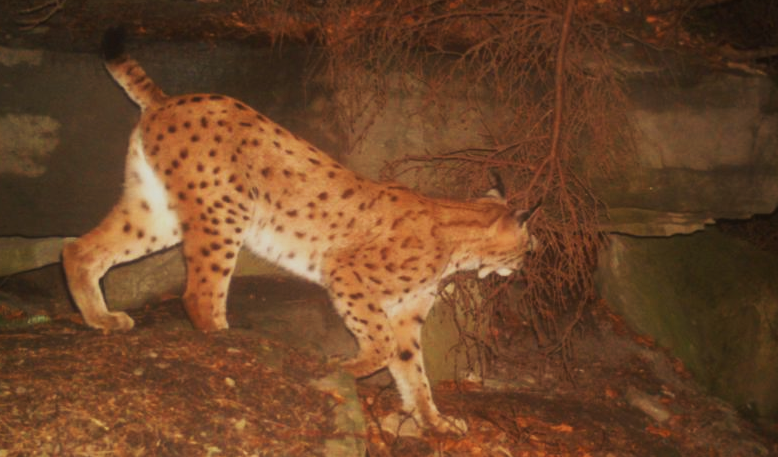


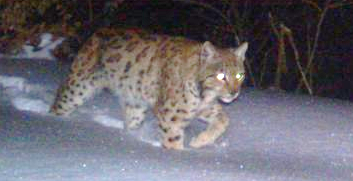

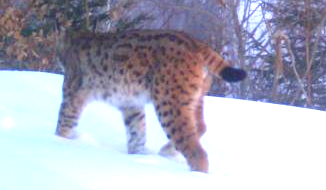
Job, ♂, A, Jan-17


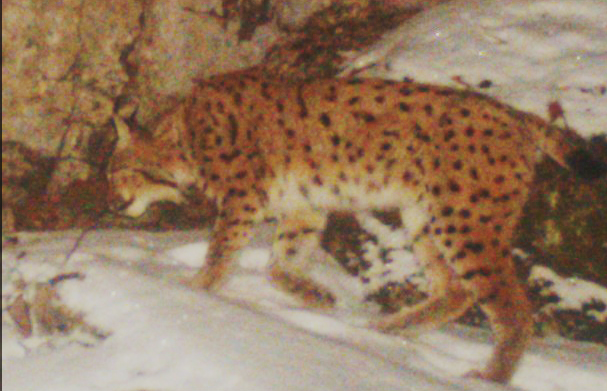

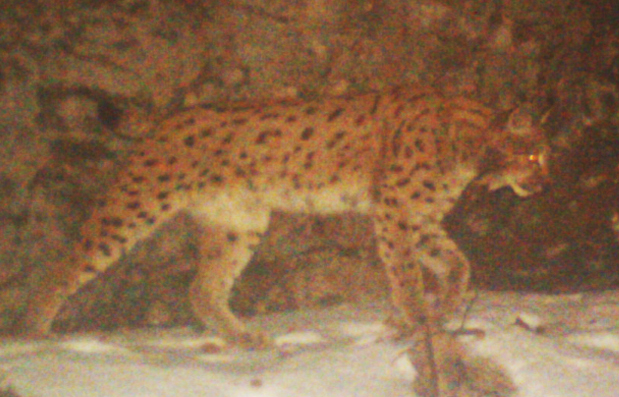
Johan, ♂ , A, Sep-17


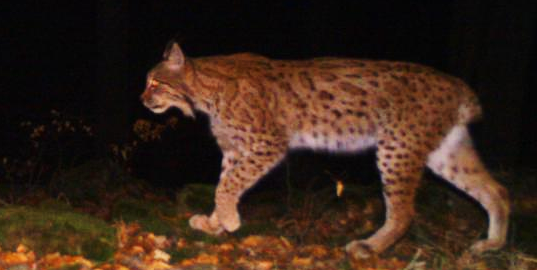

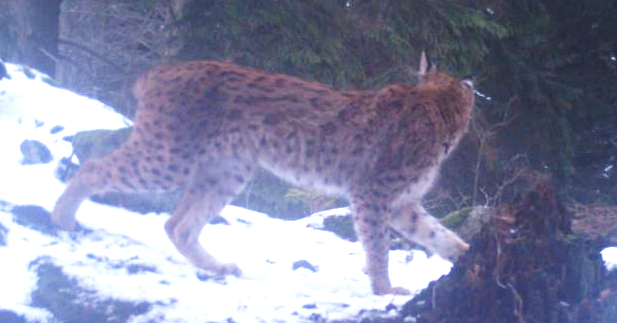
Ľubo, ♂, ND, Oct-15


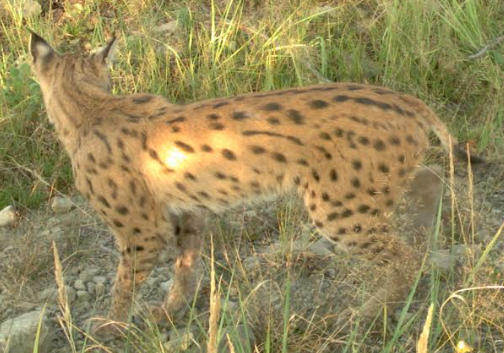

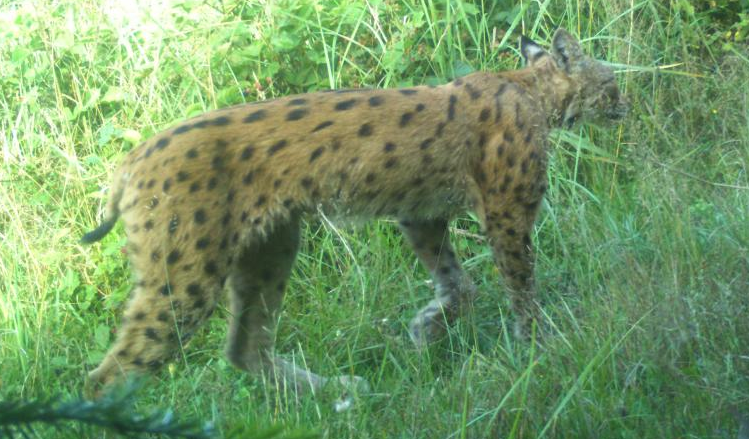
Ľudmila, ♂, A, Aug-17


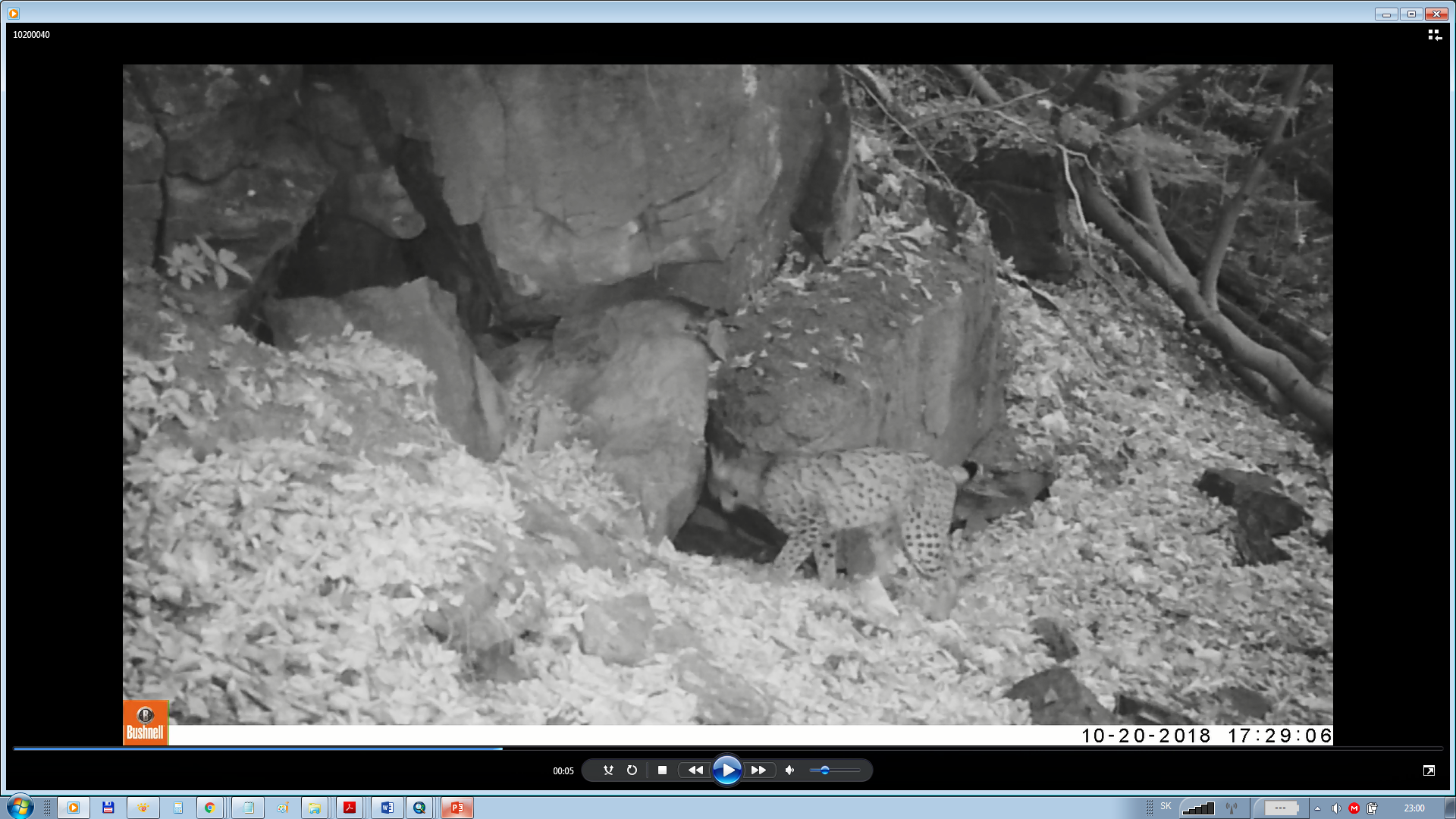

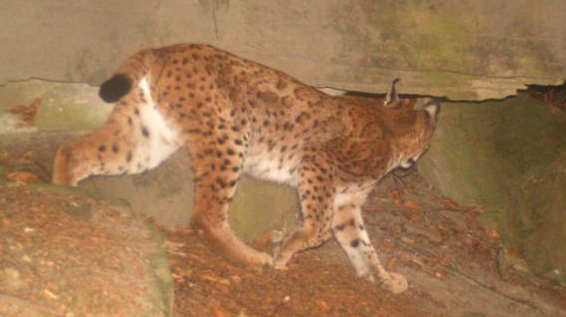
Marcela ♀*, ND, Oct-18*


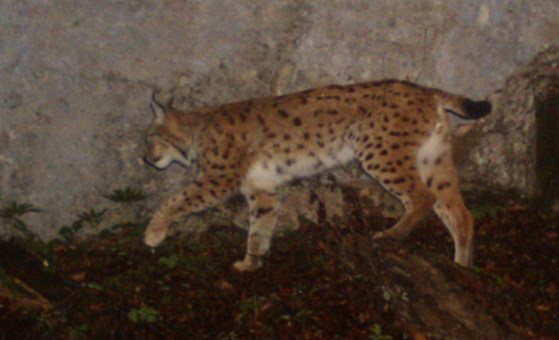

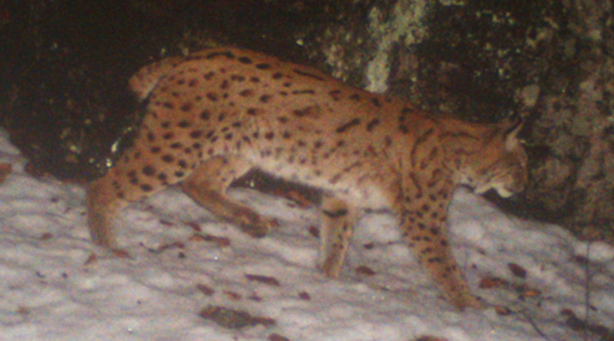
Martin, ♂, A, Nov-15


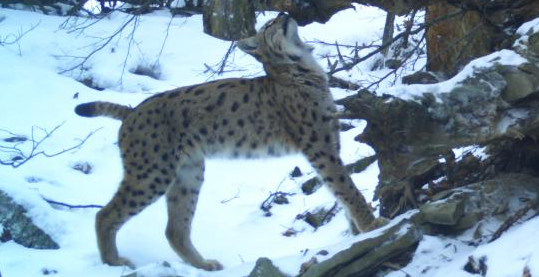

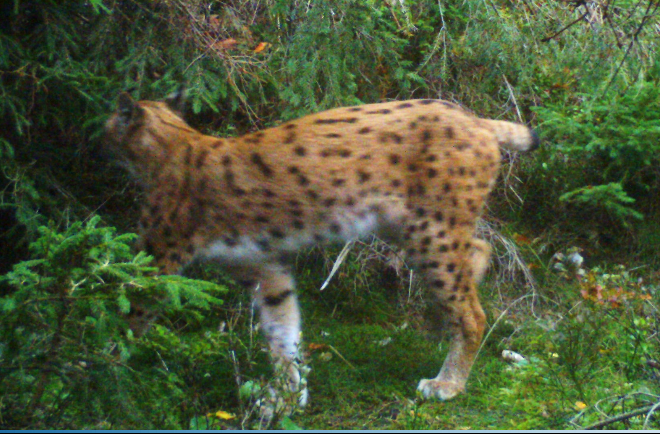
Maťo, ♂, A, Aug-17


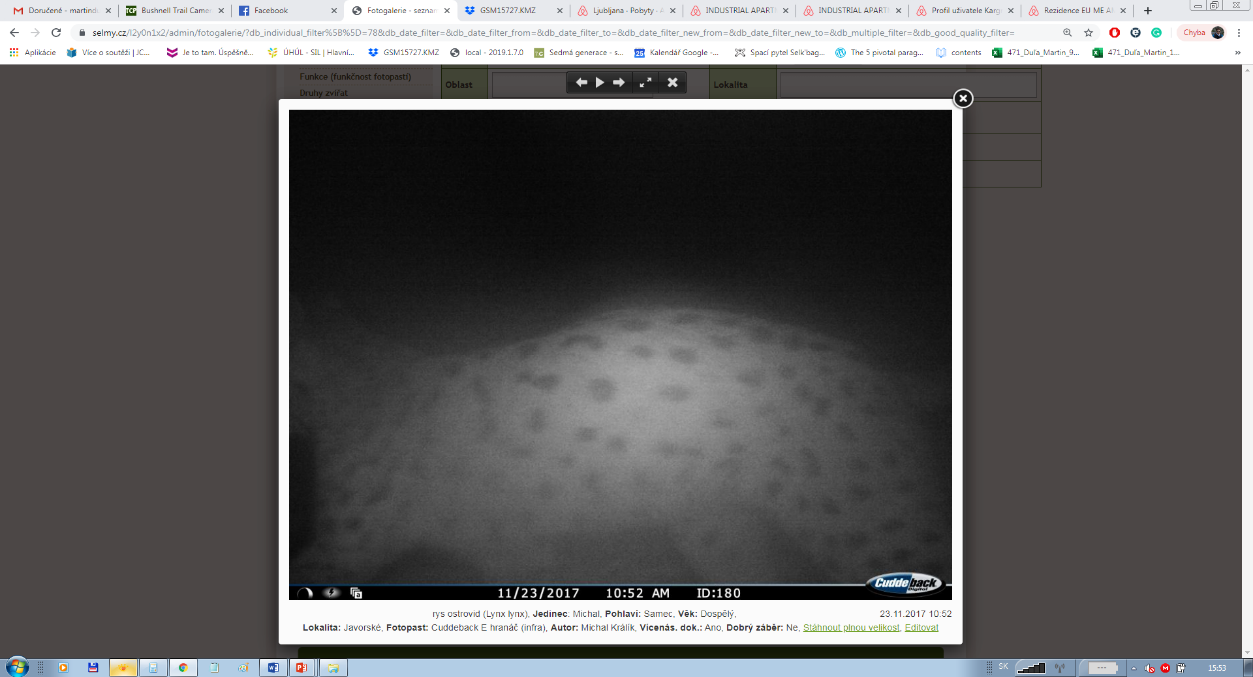

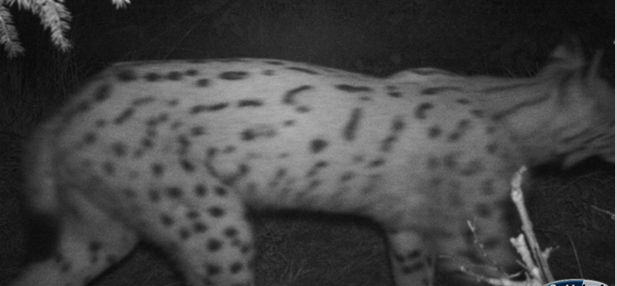
Michal, ♂, A, Sep-16


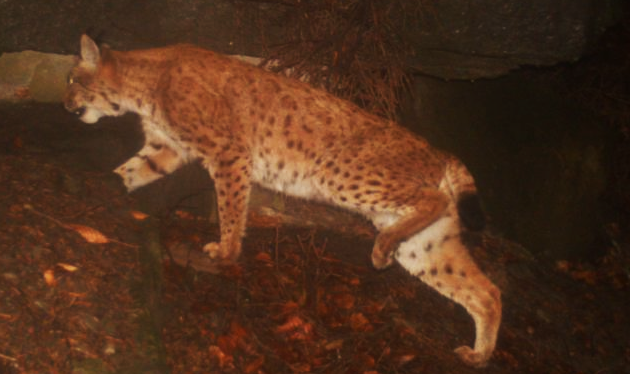

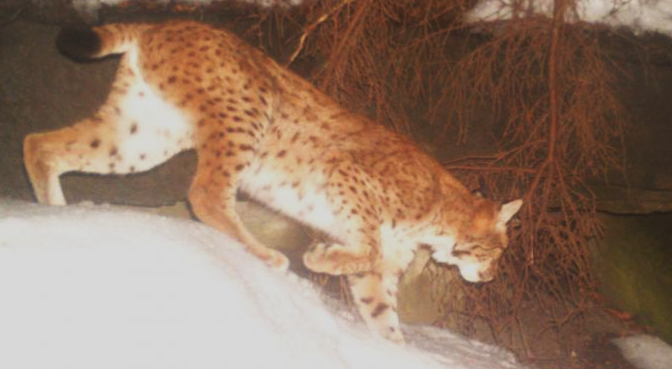
Monika, ♀*,* S, Jul-18


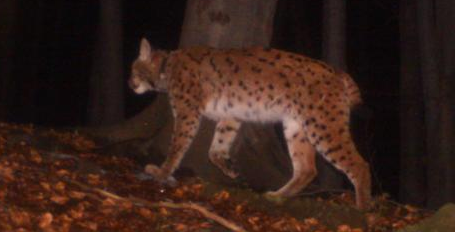

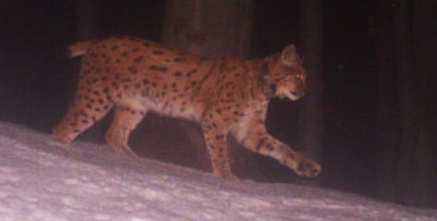
Naďa*,* ♀*,* A, Jun-15


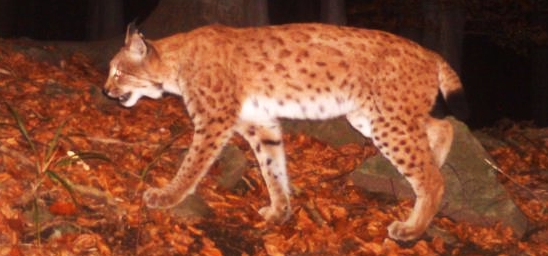

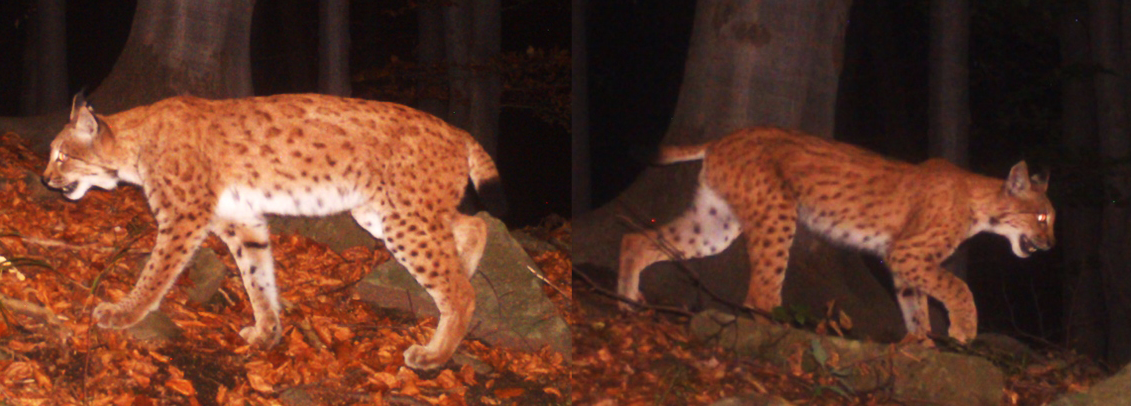
Ondrinka, ♀, A, Jul-16


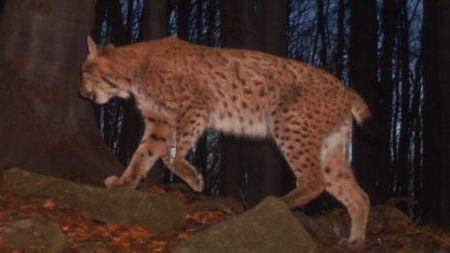

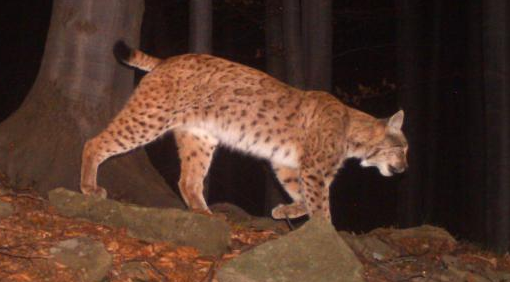
Ondro, ♂, A, May-18


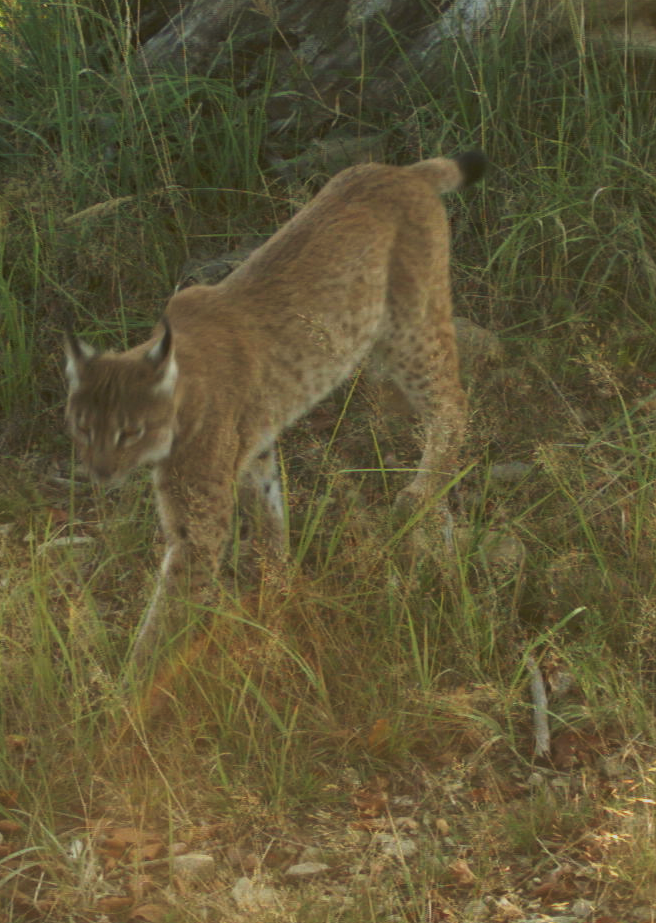

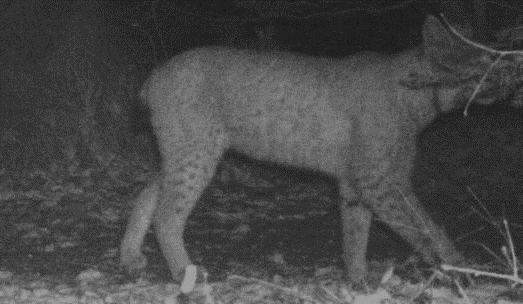
Peter, ♂, ND, Nov-19


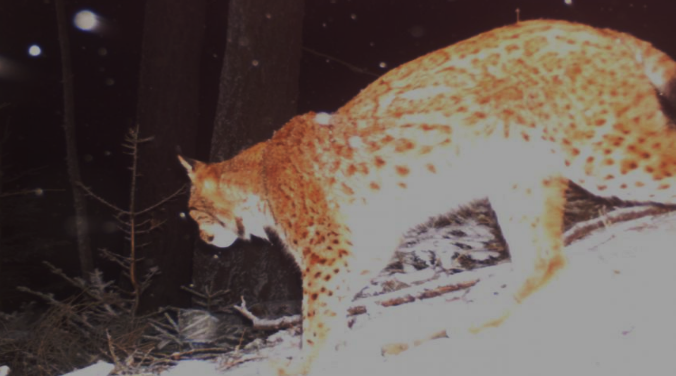

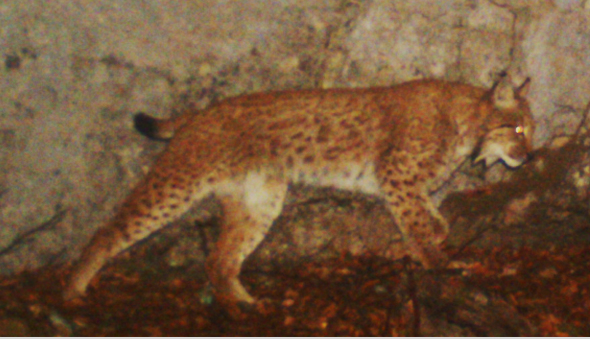
Rozeťáčik, ♂*,* A, Sep-17


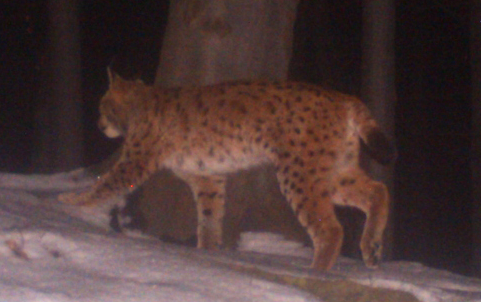

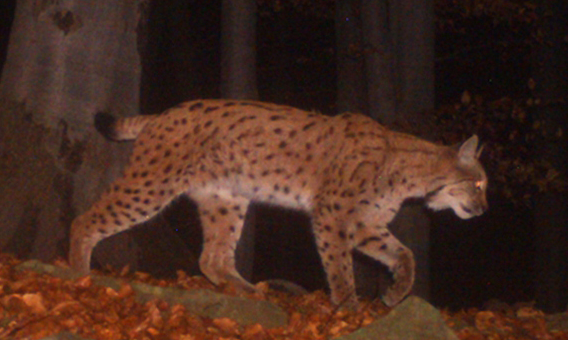
Stijn, ♂, A, Oct-16


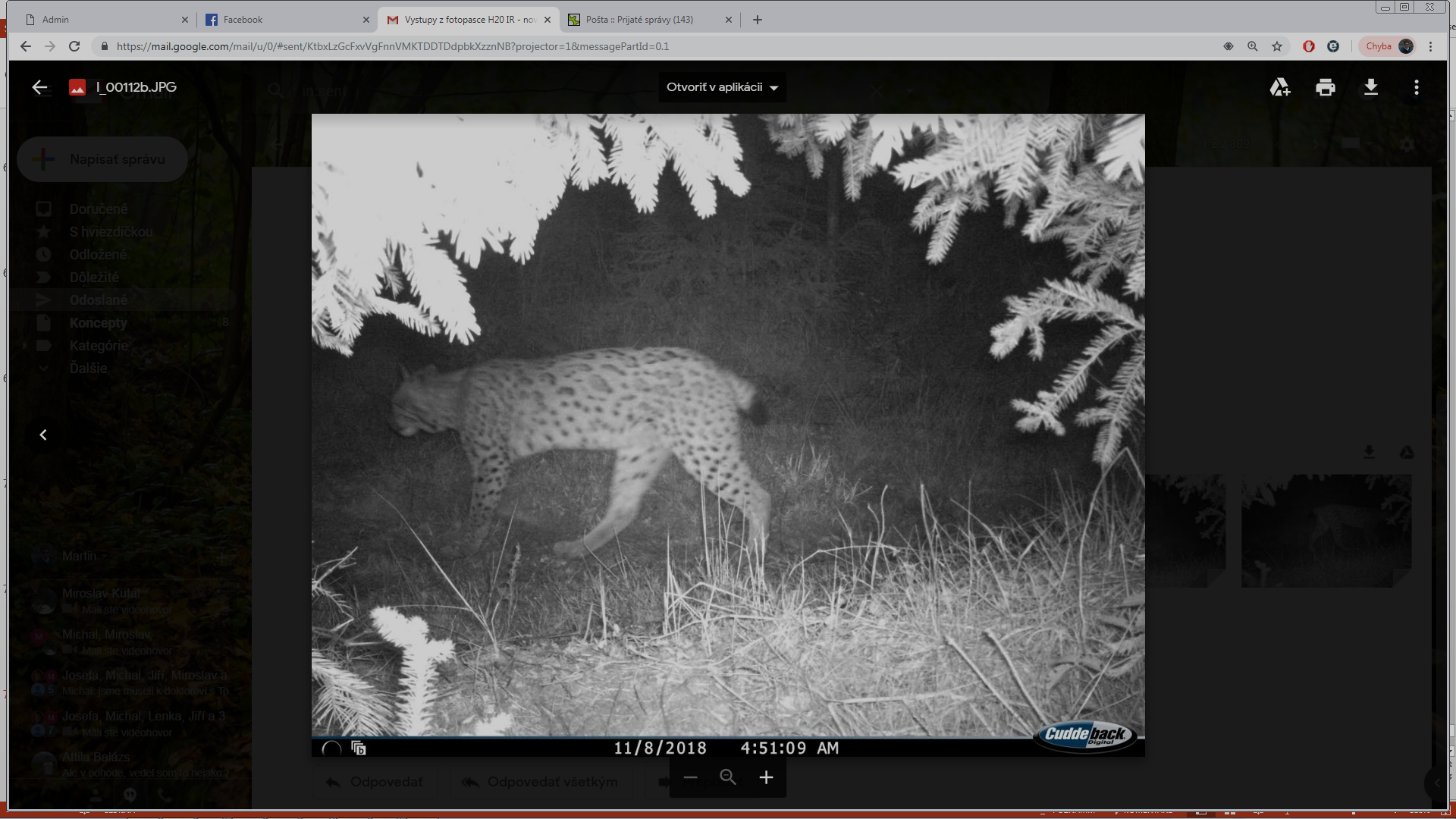

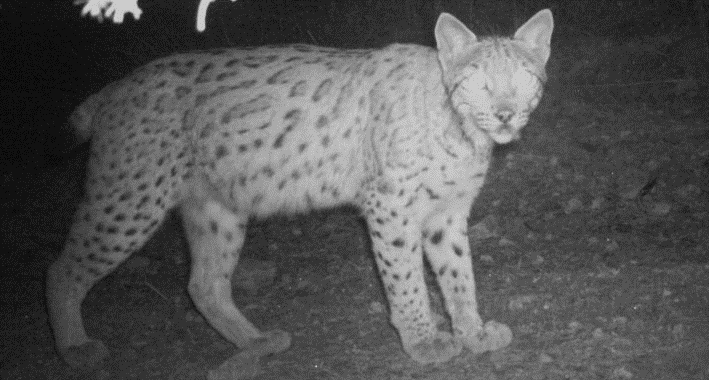
Tichomír, ♂, A, Oct-18


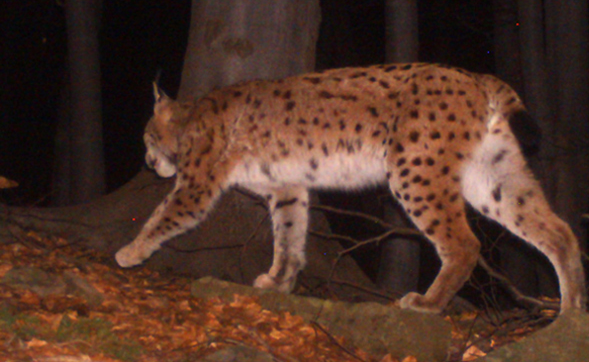

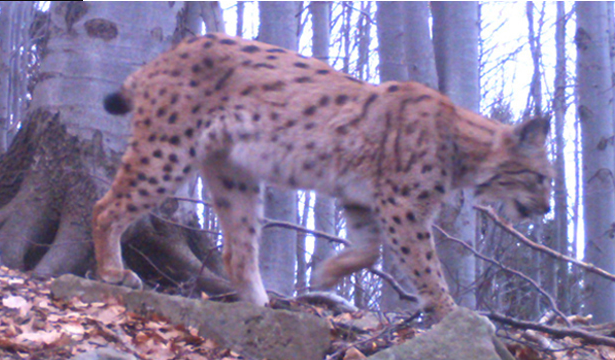
Vlado, ♂, A, Feb-17


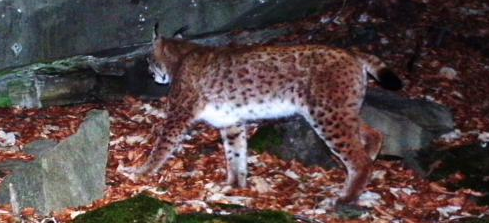

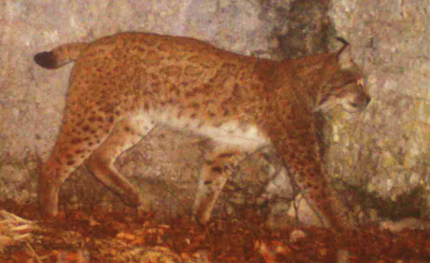
Vratko, ♂, A, Nov-14


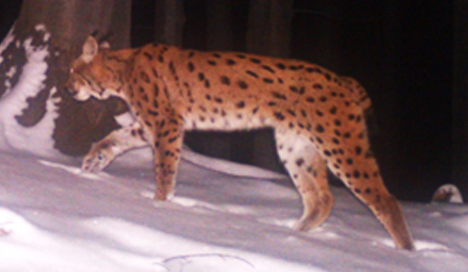
Zoja, ♀*,* A, Sep-13

No name 2, ND, S/A, Jan-18

No name 7, ND, S, Oct-15

No name 6, ND, ND, Dec-19

No name 3, ♂, ND, Oct-19

No name 4, ND*,* ND, Sep-19

No image of „R“ body site obtained

No Name 5, ND, ND, Nov-19

No image of „R“ body site obtained

(only one capture of individual)

No Name 1, ND, ND, Nov-16

No image of „R“ body site obtained

(only one capture of individual)

**References:**

Choo, YR., Kudavidanage, EP., Amarasinghe, TR., Nimalrathna, T., Chua, MAH., & Webb, EL. Best practices for reporting individual identification using camera trap photographs. Global Ecology and Conservation (24): e01294 (2020).

Stanley, TR. & Richards, J. CloseTest: A program for testing capture–recapture data for closure [Software Manual]. (2004).
